# Supplementary material for: Facade-Based Bicelles as a New Tool for Production of Active Membrane Proteins in a Cell-Free System
Source: Int J Mol Sci. 2023 Oct 3;24(19):14864. doi: 10.3390/ijms241914864 (PMC10573531; doi:10.3390/ijms241914864)
Supplement: Supplementary file 1 [file ijms-24-14864-s001.zip › GoncharukMV_Facades_CF-Blots.pdf]

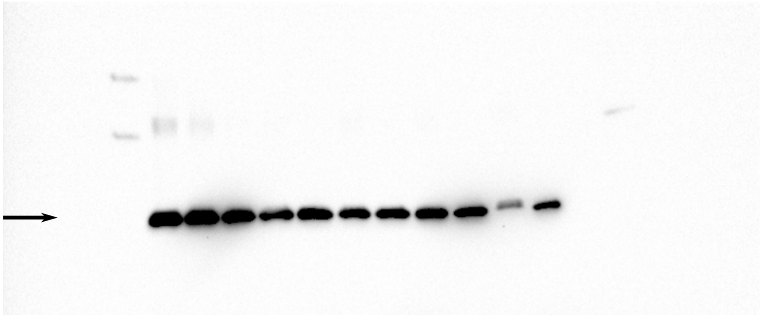

#91 M T T S T S T S T S ESR T S M
53-1-3 53-5-2 53-13 53-14 53-15 53-24

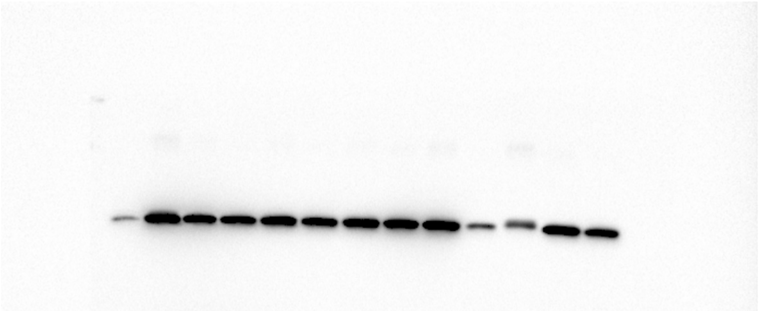

#92 M T T T S T S T S T S ESR T S S
50-18-1 50-1-4 50-7-2 50-8-1 50-15-1 50-16-1 50-17-1 50-18-1

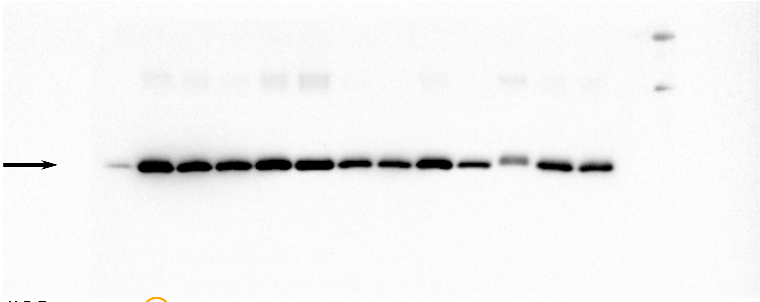

#93 T T T S T S T S T S ESR T S S M
51-18-1 51-1-4 51-6-2 51-8-1 51-15-1 51-16-1 51-17-1 51-18-1

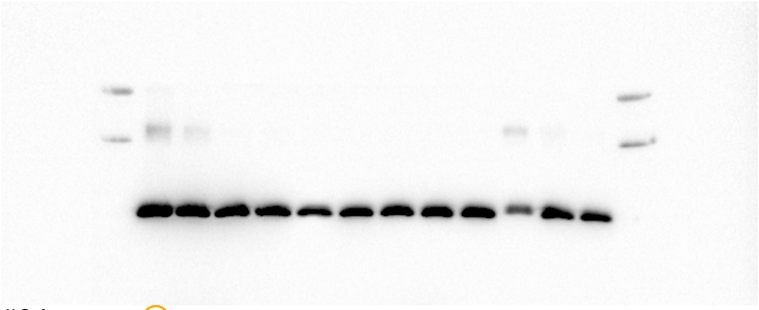

#94 M T T S T S T S T S ESR T S M
53-1-4 53-5-3 53-16 53-17 53-18 53-3

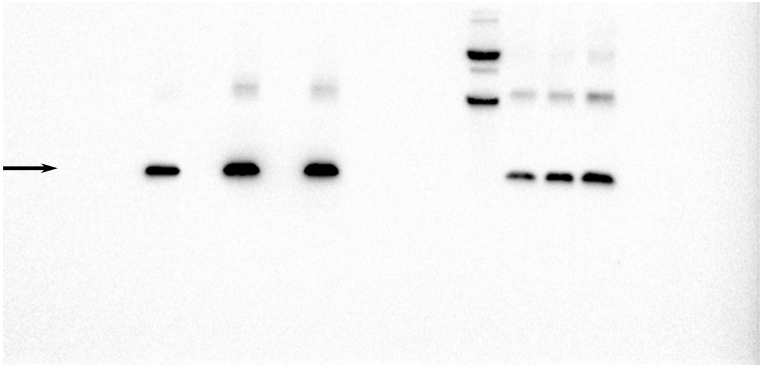

#100 T S T S T S M ESR
22-1-30 22-2-30 22-5-30

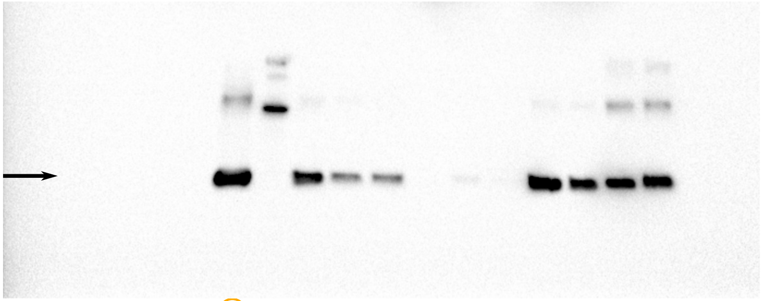

#102 T M T S T S T S T S ESR
22-1-33 22-6-33 22-7-33 22-8-33 22-9-33

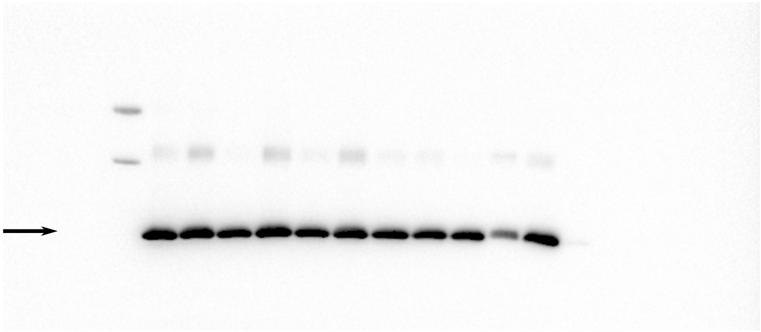

#81 M T T S T S T S T S ESR T S S
51-1-1 51-4 51-10 51-11 51-21 51-22 51-24

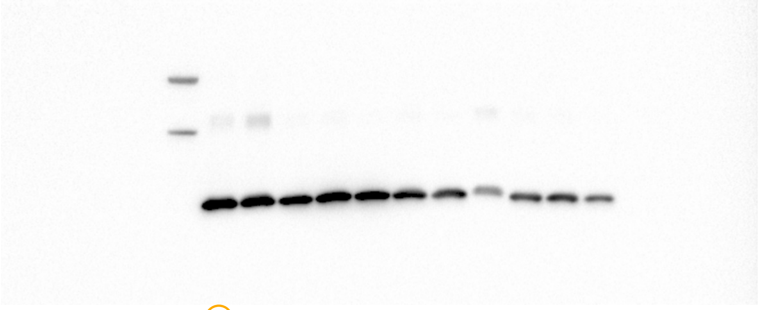

#82 S M T T S T S T S ESR T S T
51-23 51-1-2 51-4-1 51-12 51-13 51-14 51-23

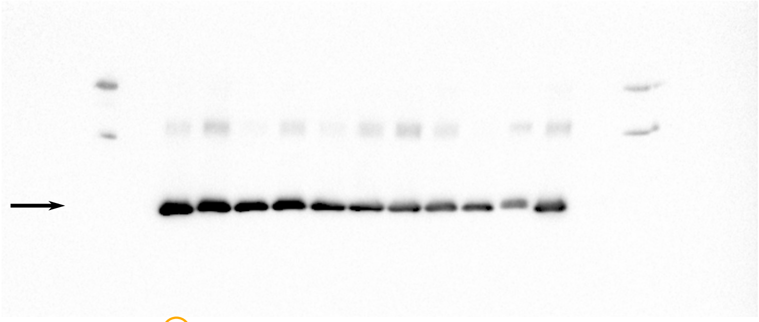

#83 M T T T S T S T S T S ESR T S M
51-24-1 51-1-3 51-4-2 51-6-1 51-8 51-19 51-20

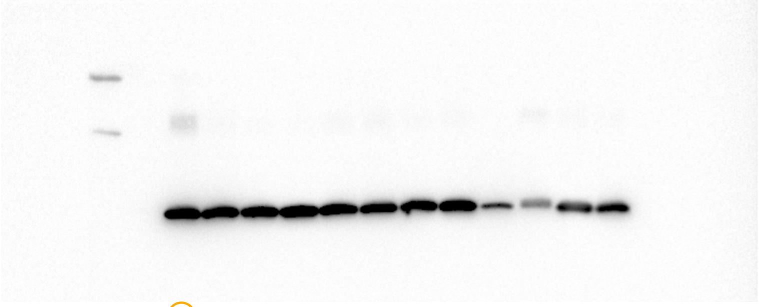

#84 M S T T S T S T S T S ESR T S
52-1 52-6 52-8 52-16 52-17 53-18

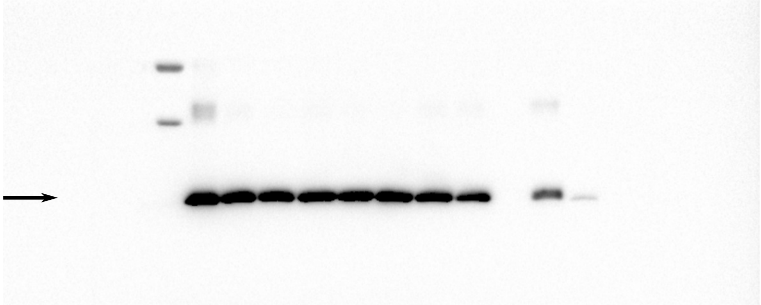

#85 M T T S T S T S T S ESR T S
52-1-1 52-5 52-6-1 52-8-1 52-20 52-19

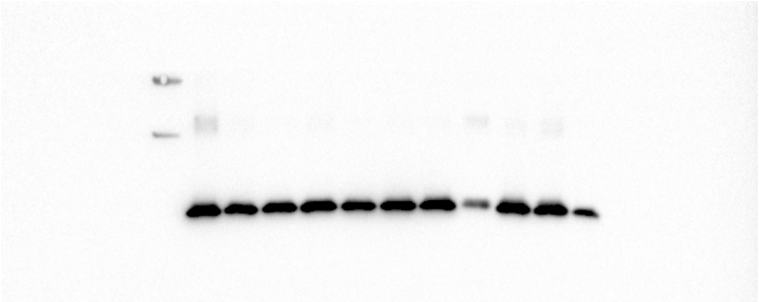

#86 S M T T S T S T S ESR T S T
52-21 52-1-2 52-5-1 52-10 52-11 52-12 52-21

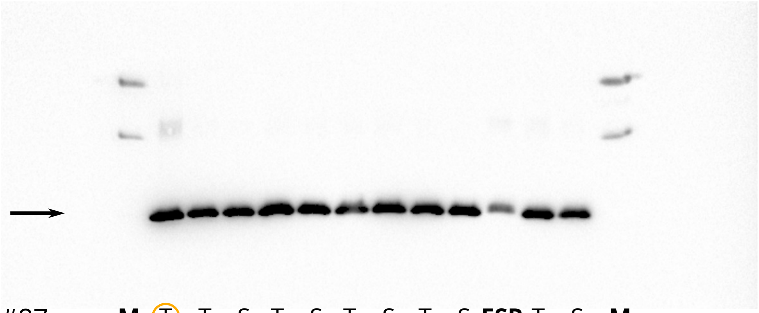

#87 M T T S T S T S T S ESR T S M
52-1-3 52-5-2 52-13 52-14 52-15 52-3

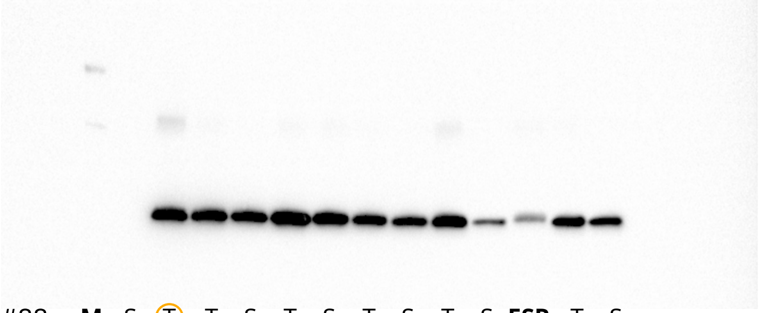

#88 M S T T S T S T S T S ESR T S
53-1 53-6 53-8 53-19 53-20 53-21

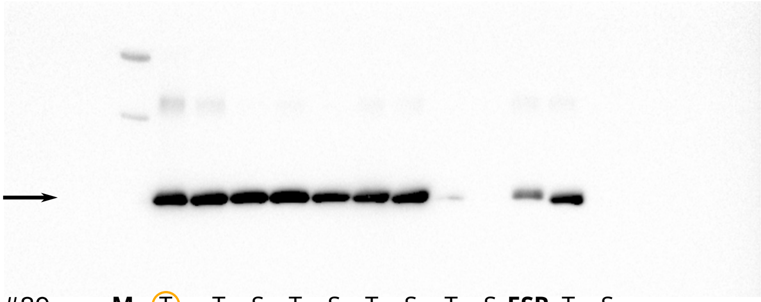

#89 M T T S T S T S T S ESR T S
53-1-1 53-5 53-6-1 53-8-1 53-22 53-23

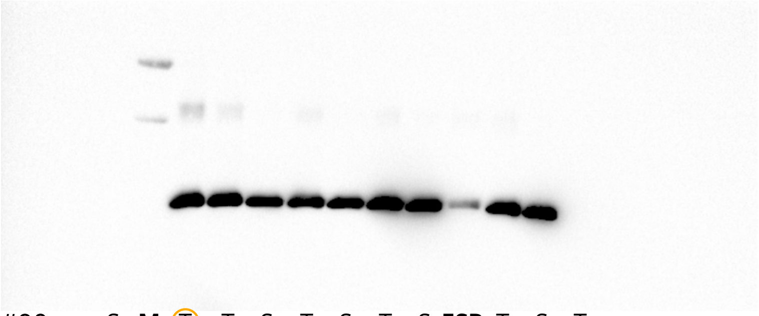

#90 S M T T S T S T S ESR T S T
53-25 53-1-2 53-5-1 53-10 53-11 53-12 53-25

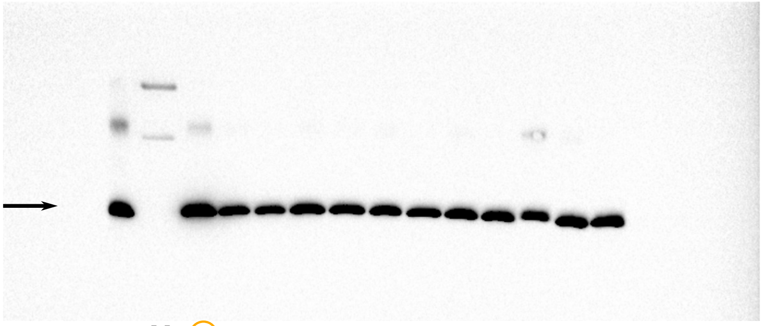

#71 M T T S T S T S T S ESR T S S
42-2-2 42-3-2 42-4-2 42-9 42-10 42-11 42-2-2

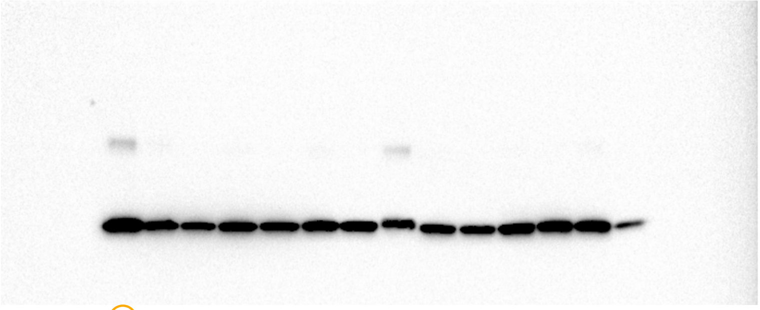

#72 M T T S T S T S ESR T S T S T S
42-2-3 42-3-3 42-4-3 42-5-2 42-15 42-17 42-14

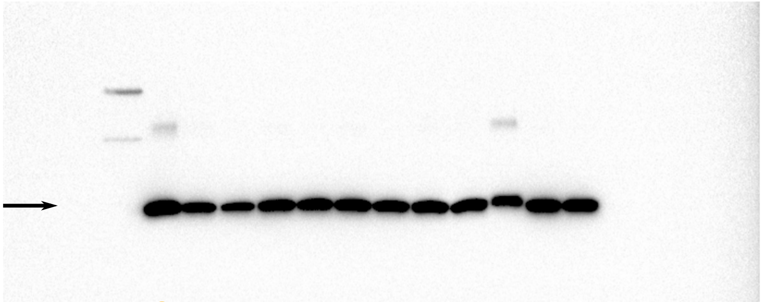

#73 M T T S T S T S T S ESR T S
42-2 42-3 42-4 42-5 42-18 42-19

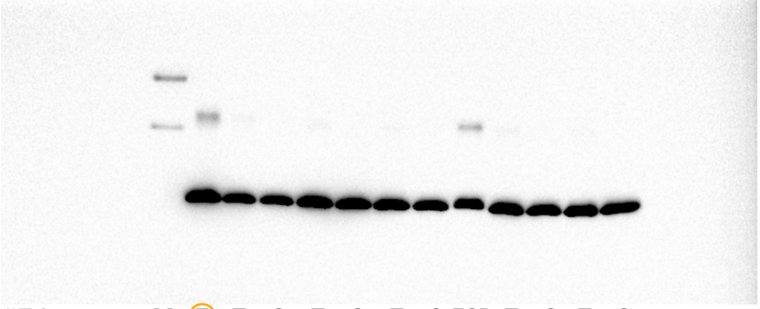

#74 M T T S T S T S ESR T S T S
42-2-4 42-3-4 42-4-4 42-5-3 42-7 42-8

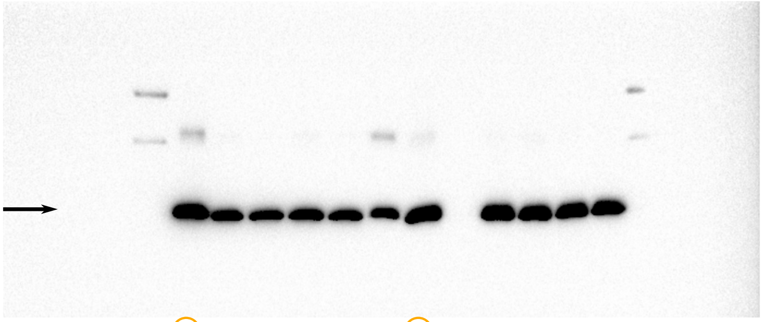

#75 M T T S T S ESR T S T S T S M
42-2-5 42-3-5 42-6 31-13 31-14 31-15

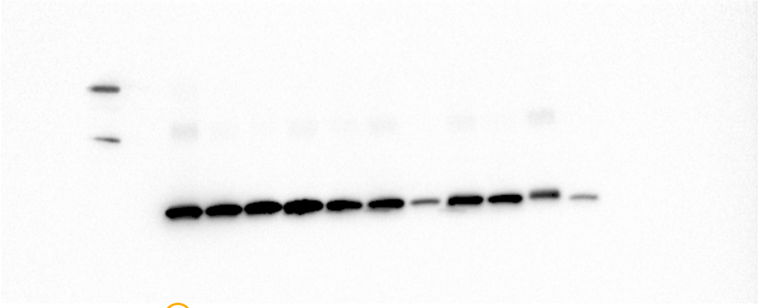

#76 M S T T S T S T S T S ESR T S
50-1 50-7 50-15 50-16 50-17 50-18

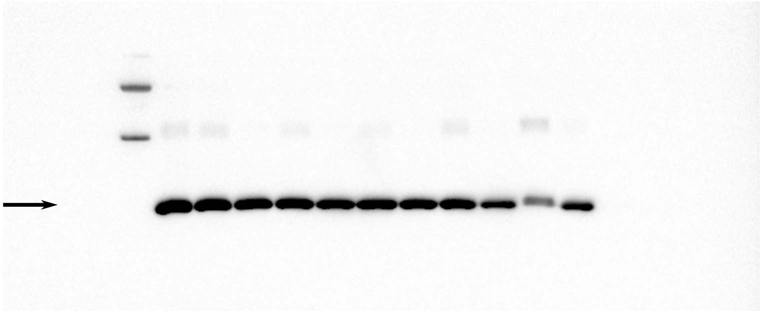

#77 M T T S T S T S T S ESR T S
50-1-1 50-4 50-10 50-11 50-21 50-22

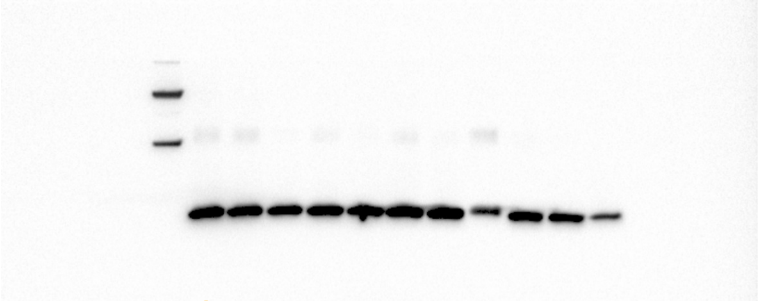

#78 S M T T S T S T S ESR T S T
50-23 50-1-2 50-4-1 50-12 50-13 50-14 50-23

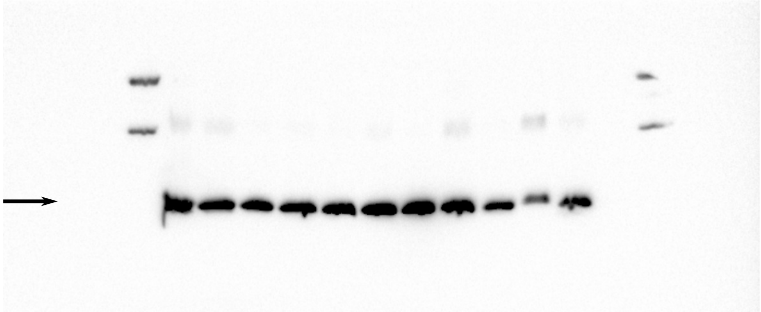

#79 M T T S T S T S T S ESR T S M
50-1-3 50-4-2 50-7-1 50-8 50-19 50-20

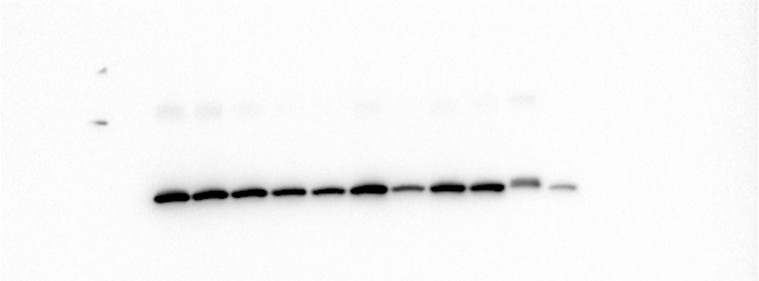

#80 M S T T S T S T S T S ESR T S
51-1 51-6 51-15 51-16 51-17 51-18

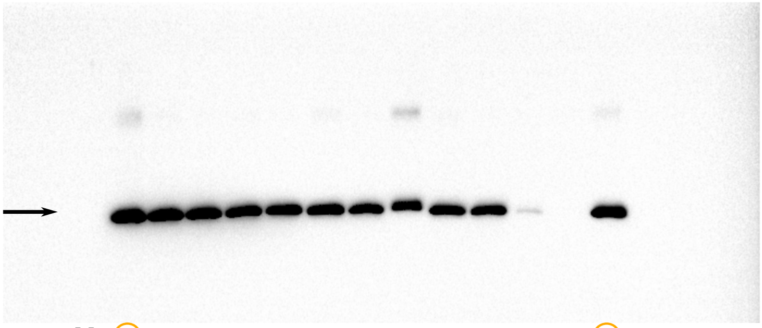
#61
M T T S T S T S ESR T S T S T S
39-1-7 39-9-2 39-12-2 41-2 41-4 41-27 41-1

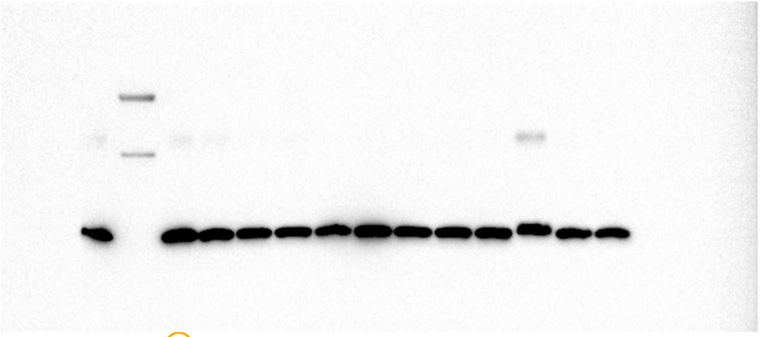
#62
M T T S T S T S T S ESR T S
41-1-1 41-2-1 41-3 41-28 41-29 41-30

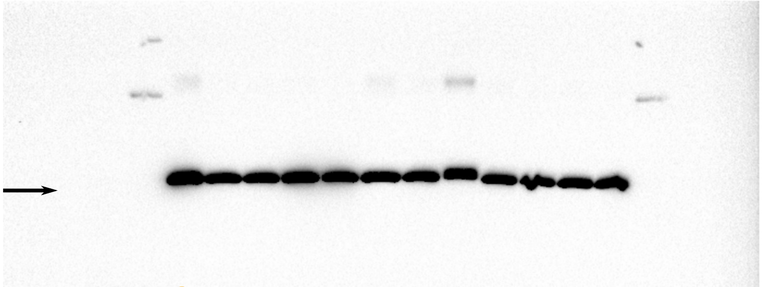
#63
M T T S T S T S ESR T S T S M
39-1-8 39-3-7 39-6-3 39-13-2 39-14-2 39-15-2

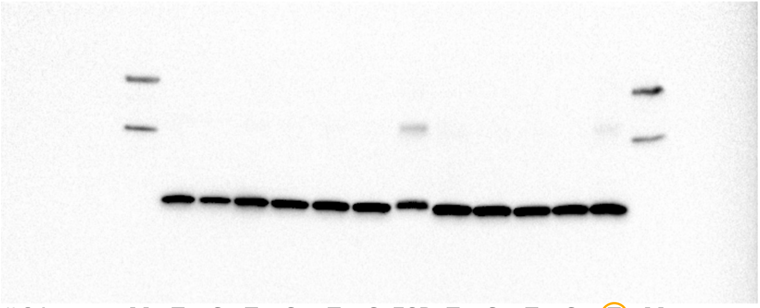
#64
M T S T S T S ESR T S T S T M
40-6 40-7 40-10 40-11 40-12 40-5

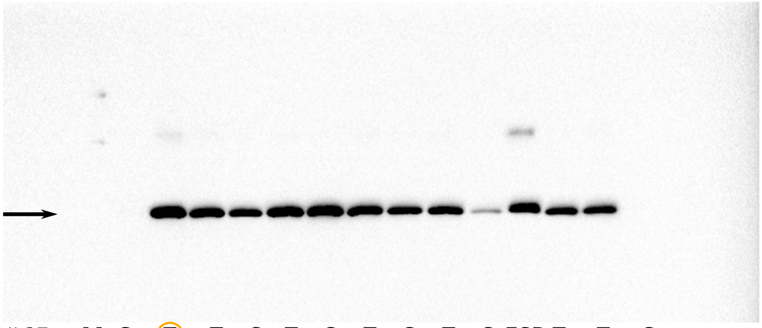
#65
M S T T S T S T S T S ESR T T S
40-19 40-5-1 40-6-1 40-7-1 40-16 40-17 40-18 40-19 40-18

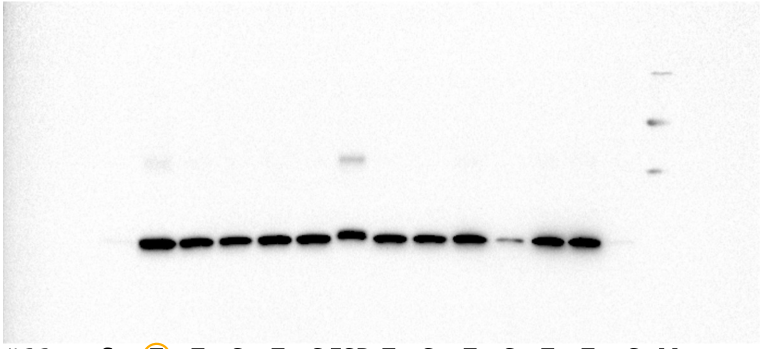
#66
S T T S T S ESR T S T S T T S M
40-23 40-5-2 40-6-2 40-7-2 40-20 40-21 40-22 40-23 40-22

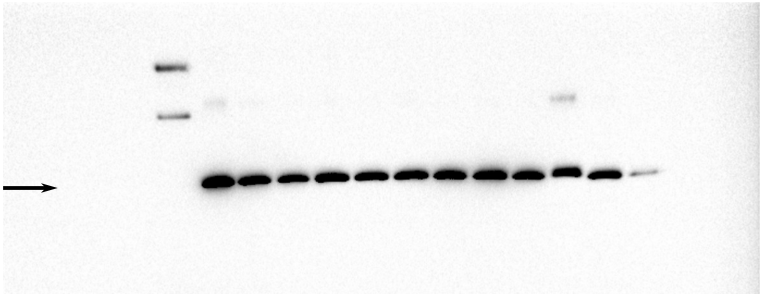
#67
S M T T S T S T S T S ESR T S
40-5-3 40-5-3 40-6-3 40-9 40-24 40-26 40-25

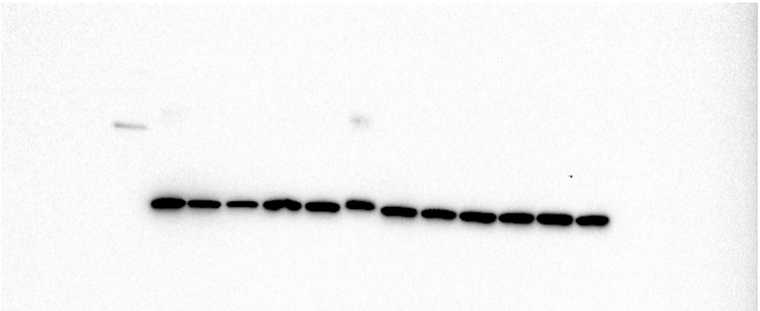
#68
M T T S T S ESR T S T S T S
40-5-4 40-6-4 40-7-3 40-13 40-14 40-15

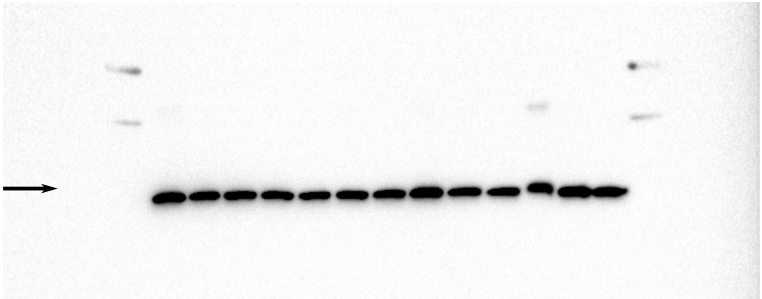
#69
M T T S T S T S T T S ESR T S M
40-5-5 40-7-4 40-8 40-9-1 41-1-2 41-3-1 41-4-1

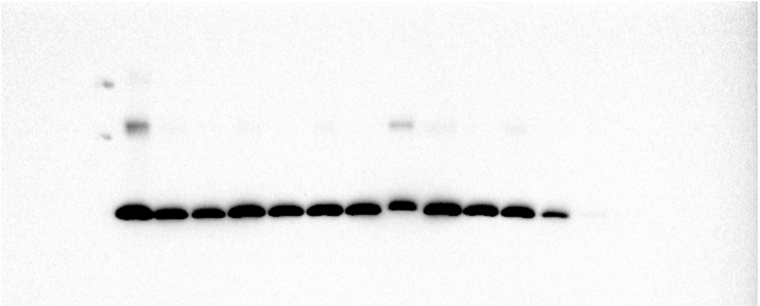
#70
M T T S T S T S ESR T S T S T S
42-2-1 42-3-1 42-4-1 42-5-1 42-12 42-13 42-16

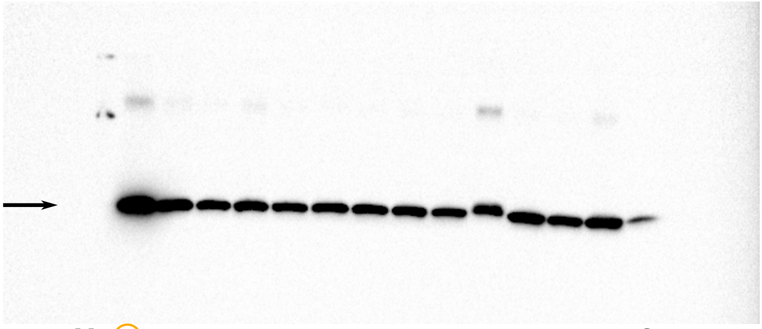

#51 M T T S T S T S T S ESR T S T S
38-1-2 38-4-2 38-6-1 38-12 38-14 38-13 38-11 38-9-1

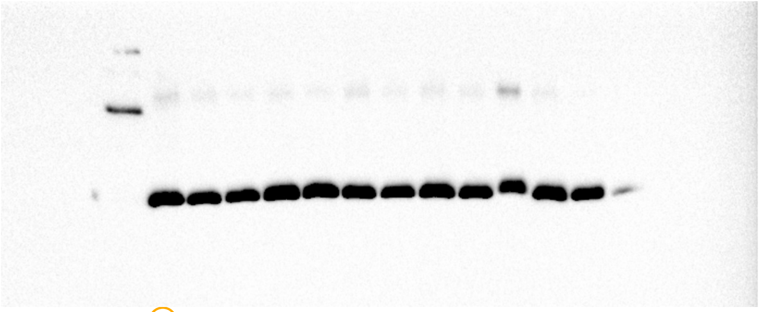

#52 M T T S T S T S T S ESR T S S
38-1-3 38-4-3 38-5 38-6-2 38-7 38-8 38-11-1

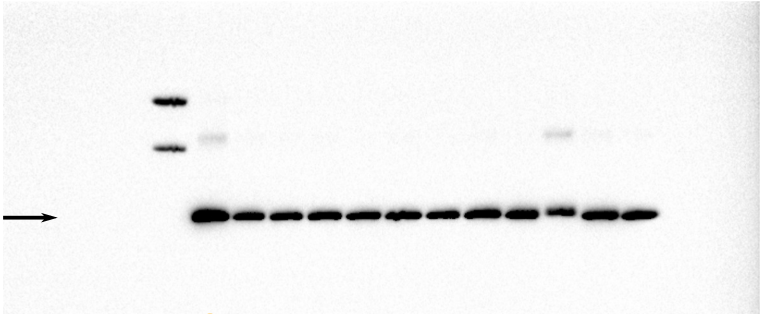

#53 S M T T S T S T S T S ESR T S
39-1 39-1 39-3 39-5 39-6 39-8 39-7

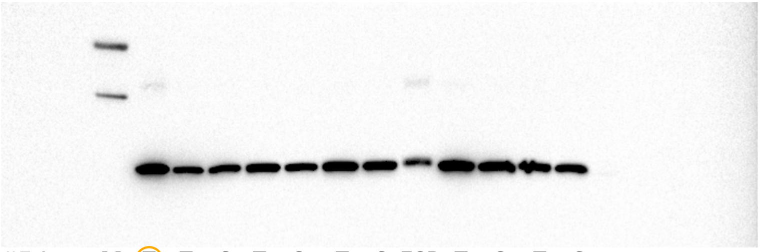

#54 M T T S T S T S ESR T S T S
39-1-1 39-3-1 39-5-1 39-9 39-10 39-11

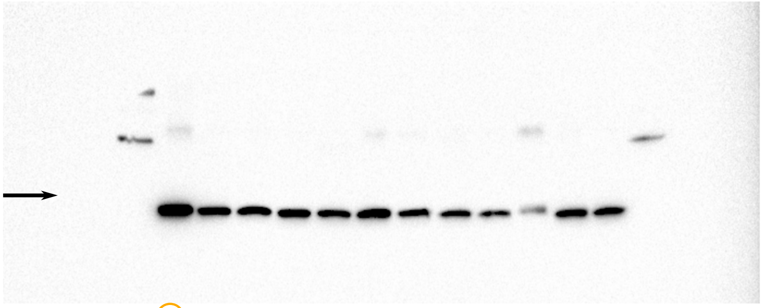

#55 M T T S T S T S T S ESR T S M
39-1-2 39-3-2 39-6-1 39-13 39-14 39-15

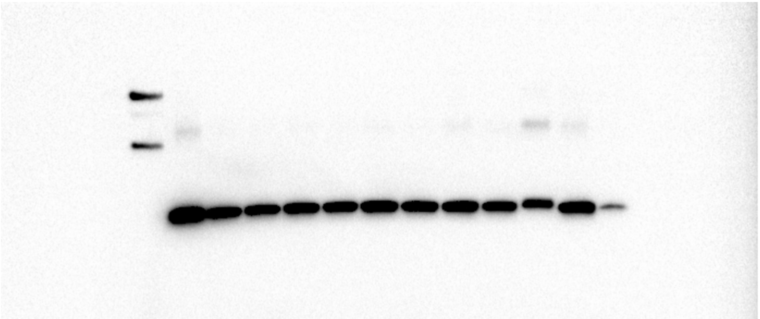

#56 M T T S T S T S T S ESR T S
39-1-3 39-3-3 39-5-2 39-12 39-18 39-19

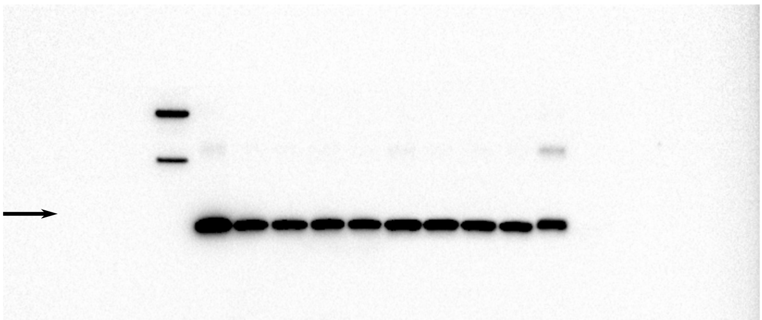

#57 S M T T S T S T S T S ESR T
39-17 39-1-4 39-3-4 39-6-2 39-16 39-5-3 39-17

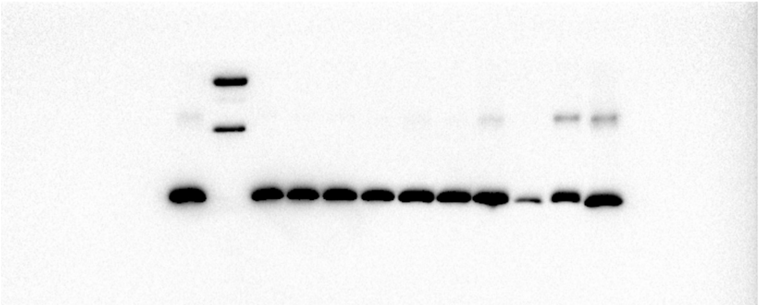

#58 S T M T S T S T S T S ESR T
39-2 39-1-5 39-3-5 39-5-4 39-20 39-21 39-2

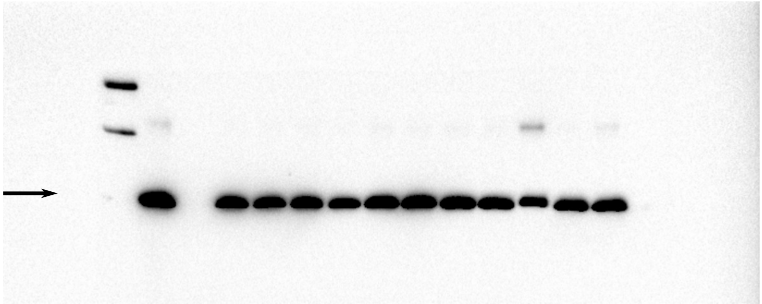

#59 M T T S T S T S T S ESR T S
39-1-6 39-3-6 39-5-5 39-22 39-23 39-24

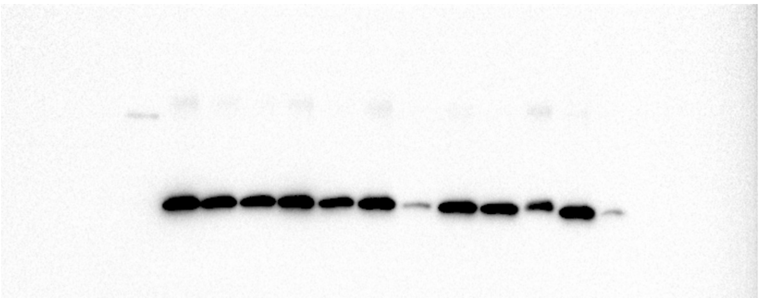

#60 M T T S T S T S T S ESR T S
38-1-4 38-4-4 38-8-1 38-9-2 38-10-2 38-11-2

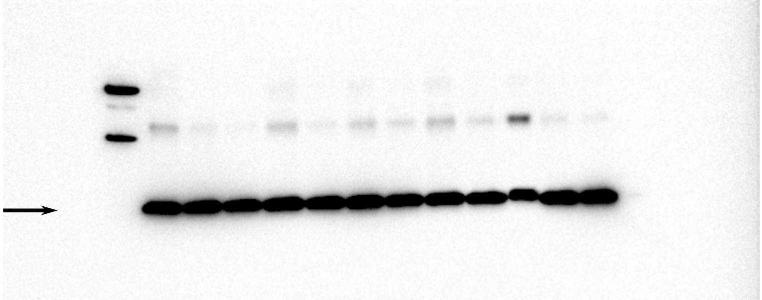

#41 M T T S T S T S T S ESR T S S
37-1 37-3 37-6 37-7 37-8 37-5 37-1

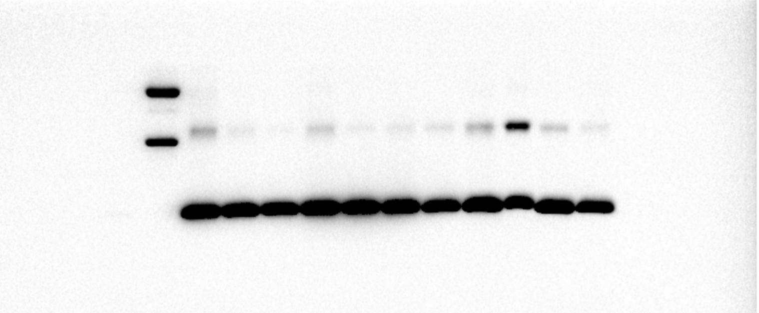

#42 S M T T S T S T S T ESR S T
37-17 37-1-1 37-3-1 37-6-1 37-9 37-13 37-13 37-17

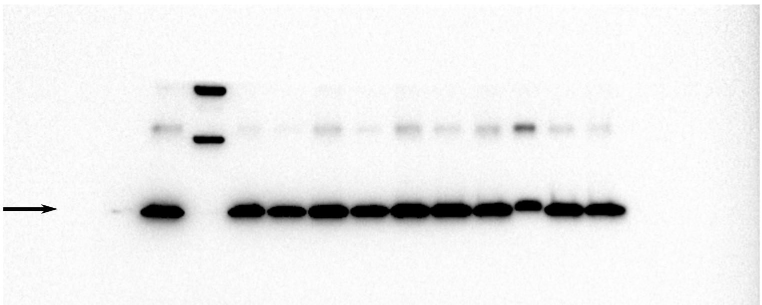

#43 S T M T S T S T S T ESR S T
37-18 37-1-2 37-3-2 37-6-2 37-11 37-12 37-12 37-18

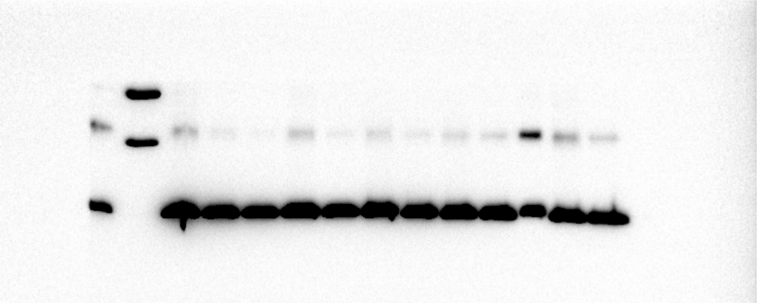

#44 M T T S T S T S T S ESR T S
37-1-3 37-3-3 37-6-3 37-19 37-20 37-16

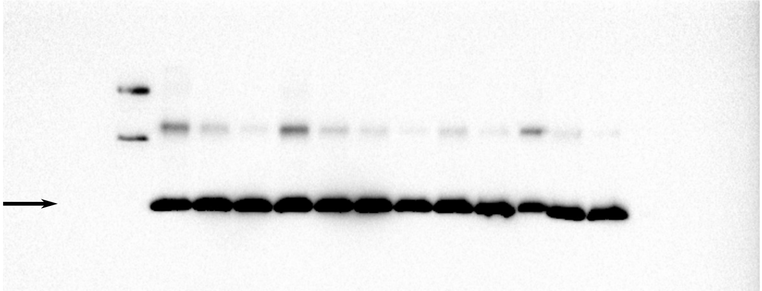

#45 M T T S T S T S T S ESR T S
37-1-4 37-3-4 37-6-4 37-10 37-14 37-15

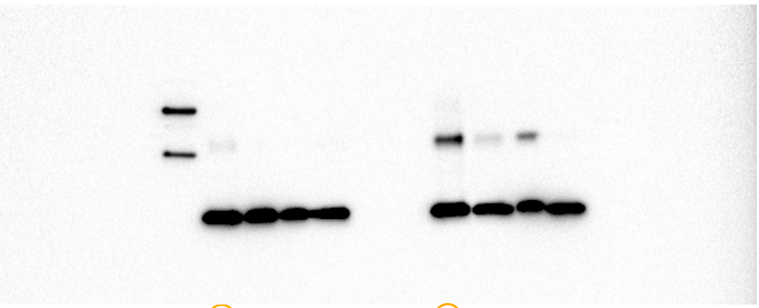

#46 M T T S T S T T T ESR S T
33-1-4 33-8-1 33-18 36-19 36-1-4 36-20-4 36-20-4 36-19

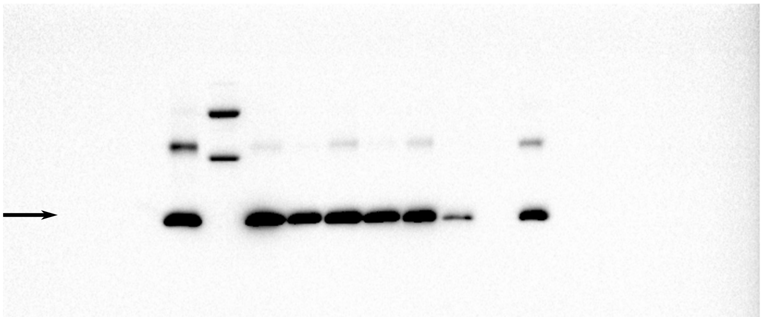

#47 S T M T S T S T S T ESR T S
36-17 36-1-5 36-20-5 36-8 36-15 36-17 36-18

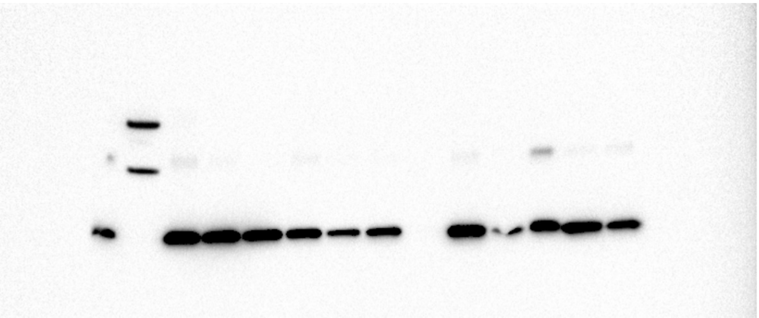

#48 M T T S T S T S T S ESR T S
33-1-5 33-8-2 33-14 33-15 33-16 33-17

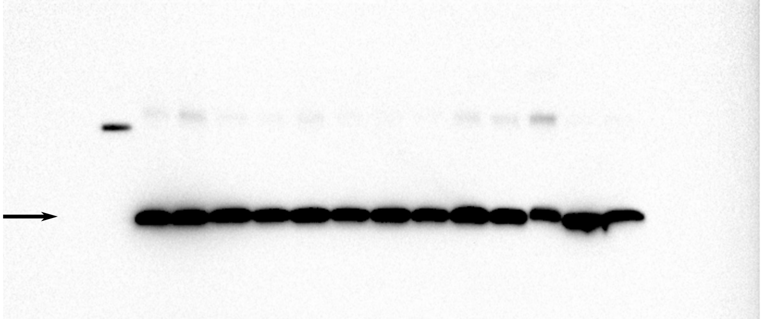

#49 M T T T S T S T S T S ESR T S
38-9 38-1 38-4 38-6 38-15 38-17 38-16

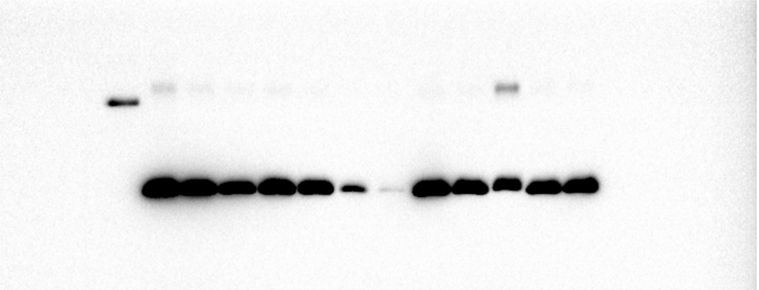

#50 M T T S T S T S T S ESR T S S
38-1-1 38-4-1 38-10 38-21 38-18 38-19 38-1-1

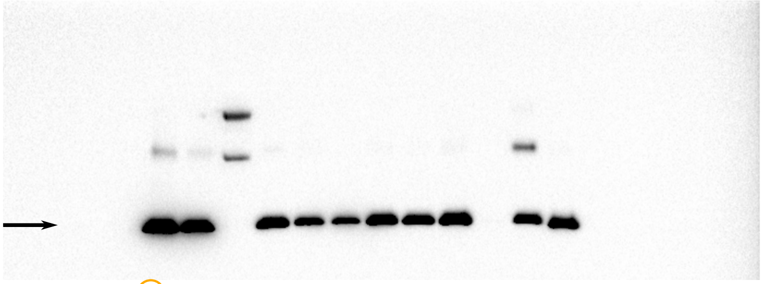

#31 T T M S T S T S T S ESR T S
34-2-2 34-20-2 34-20-2 34-6-1 34-9 34-10 34-11

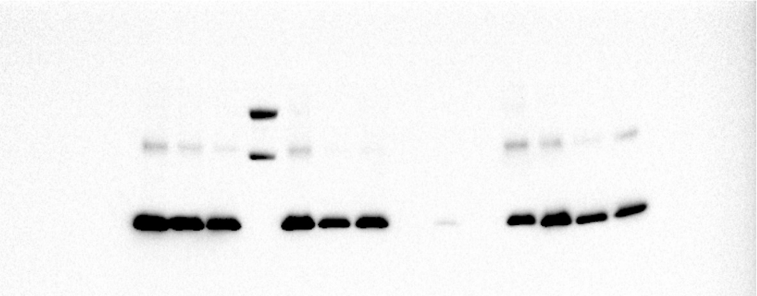

#32 T T S M T S T S T S ESR T S
34-2-3 34-20-3 34-16 34-17 34-18 34-19 34-19

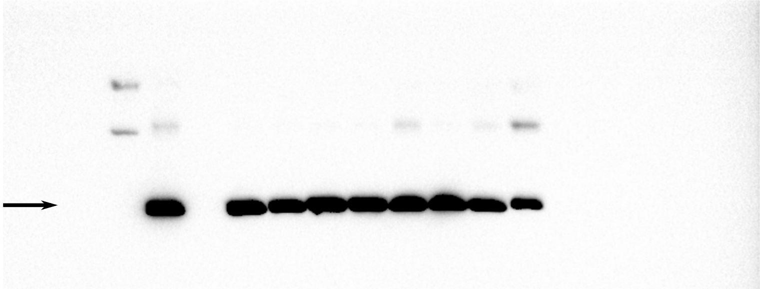

#33 M T S T S T S T S T ESR S T S
35-2 35-3 35-4 35-20 35-10 35-10 35-11

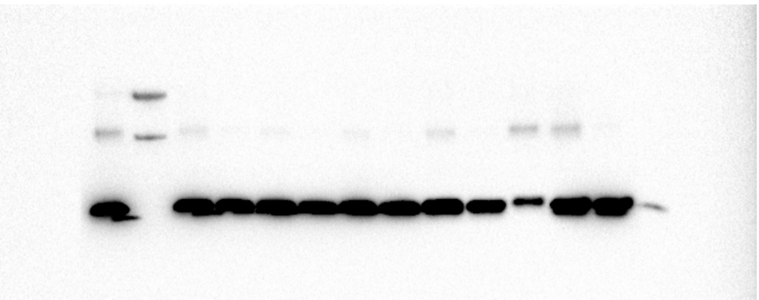

#34 T M T S T S T S T S ESR T S T
35-2-1 35-20-1 35-6 35-7 35-8 35-9 35-19

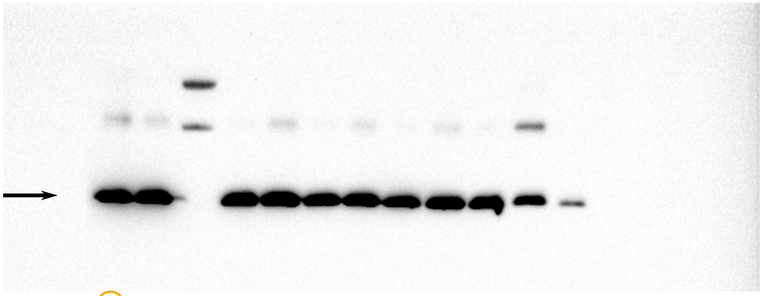

#35 T T M S T S T S T S ESR T S
35-2-2 35-20-2 35-20-2 35-12 35-13 35-14 35-18

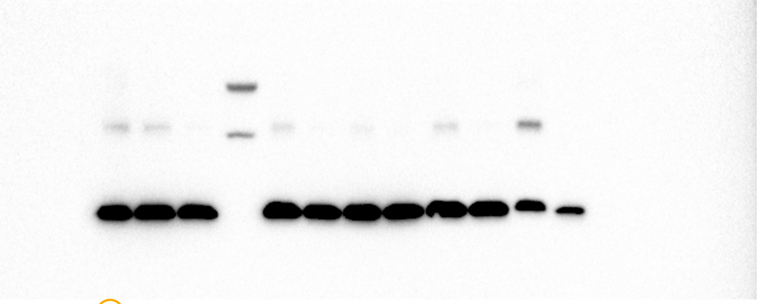

#36 T T S M T S T S T S ESR T S S
35-2-3 35-20-3 35-8-1 35-15 35-16 35-17 35-19-1

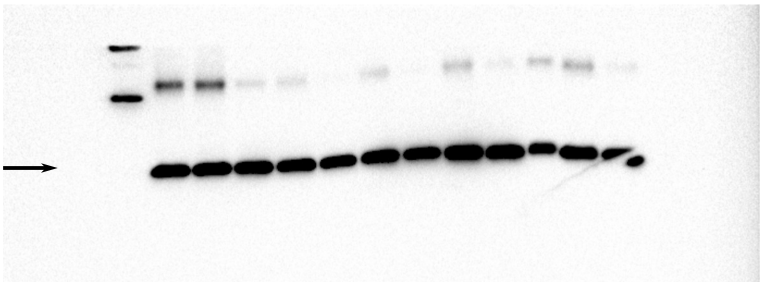

#37 M T T S T S T S T S ESR T S
36-1 36-3 36-4 36-20 36-13 36-14

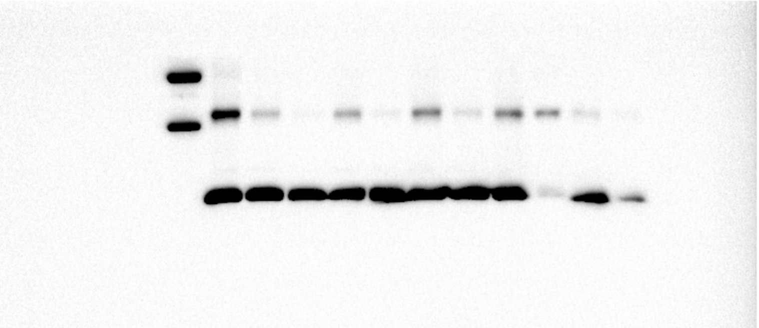

#38 S M T T S T S T S ESR
36-1-1 36-1-1 36-20-1 36-5 36-6

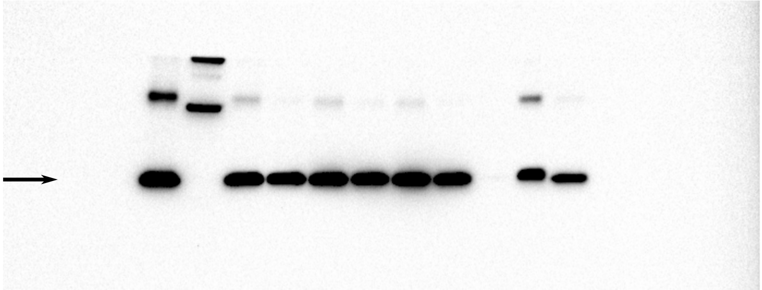

#39 S T M T S T S T S T ESR T S
36-10 36-1-2 36-20-2 36-11 36-12 36-10 36-9

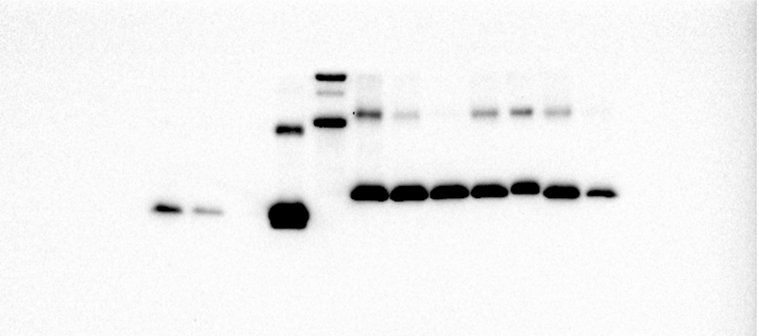

#40 M T T S ESR T S
36-1-3 36-20-3 36-16

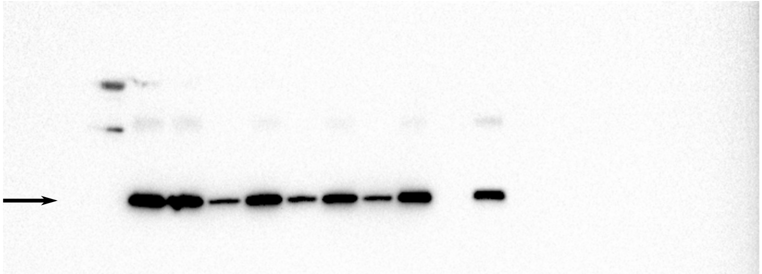

#21 M T T S T S T S ESR
28-1-2 28-7 28-8 28-9

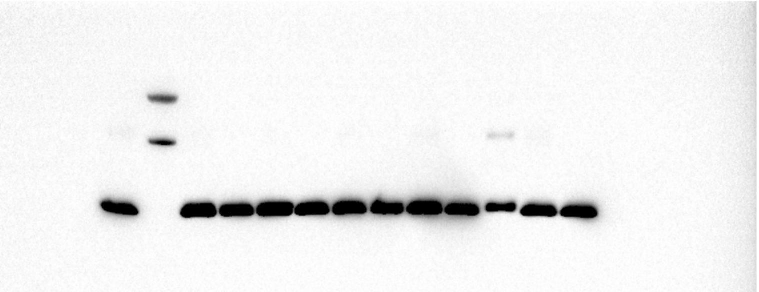

#22 T M T S T S T S T S ESR T S
29-1 29-10 29-11 29-12 29-2 29-16

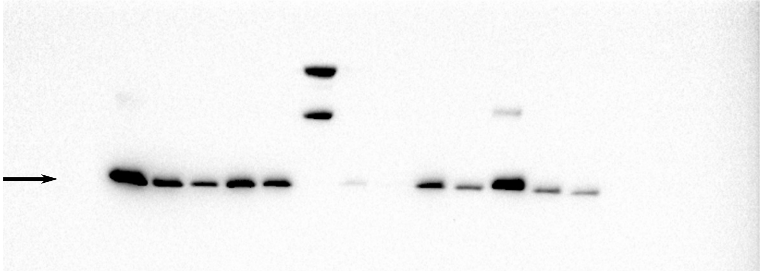

#23 T T S T S M T S T S ESR S
29-1-1 29-3 29-4 29-5 29-13 29-1-1

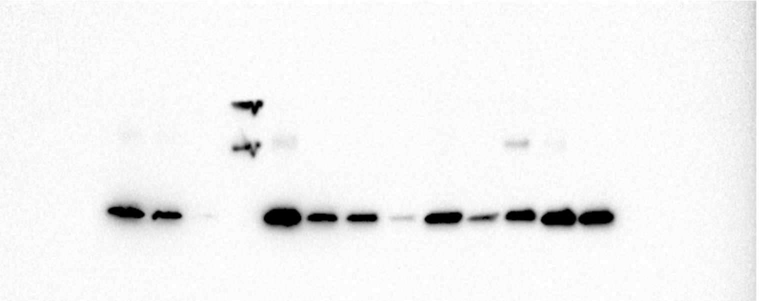

#24 T T S M T S T S T S ESR T S
29-1-2 29-6 29-7 29-8 29-9 29-15

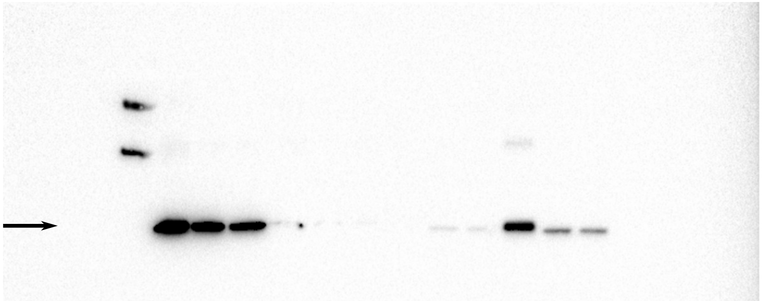

#25 M T T S T S T S T S ESR T S S
30-1 30-2 30-3 30-6 30-4 30-7 30-1

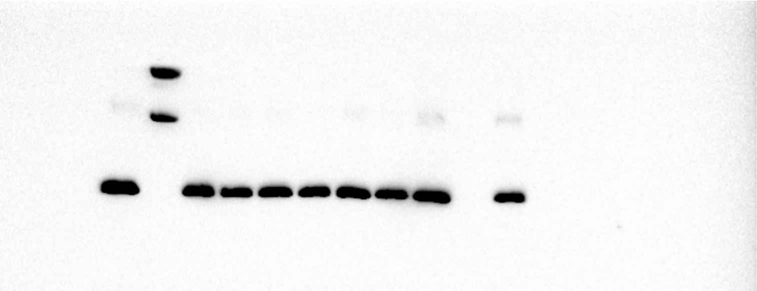

#26 T M T S T S T S T T ESR S T S
30-1-1 30-2-1 30-5 30-8 33-1 33-12 33-12 33-13

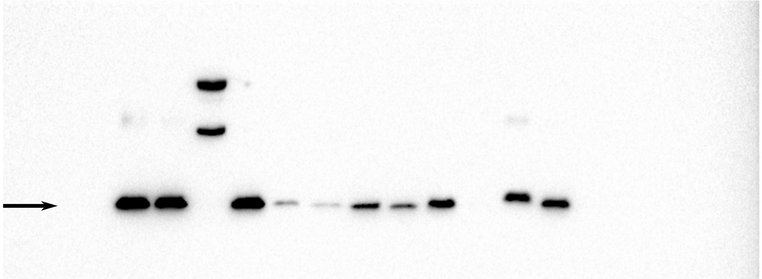

#27 T T M S T S T S T S ESR T S S
33-1-1 33-3 33-3 33-4 33-5 33-6 33-7 33-1-1

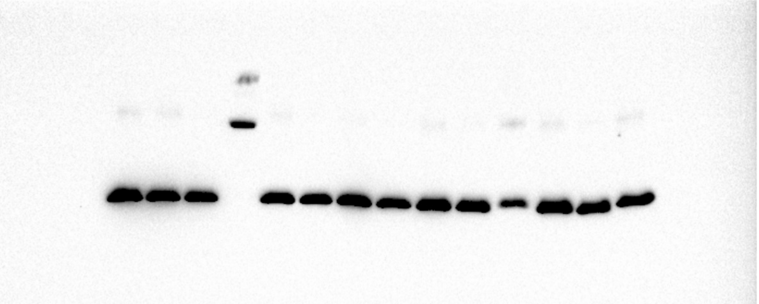

#28 T T S M T S T S T S ESR T S T
33-1-2 33-3-1 33-8 33-9 33-10 33-11 33-1-3

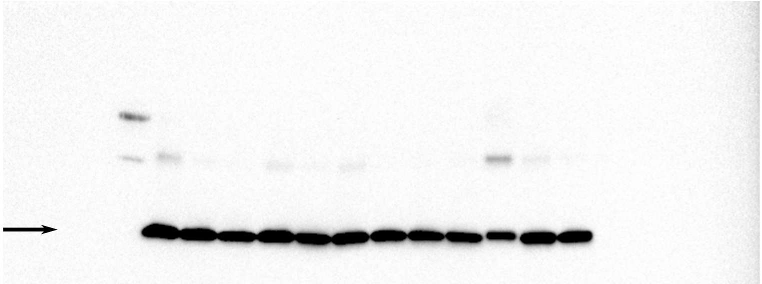

#29 M T T S T S T S T S ESR T S S
34-2 34-4 34-20 34-13 34-14 34-15 34-2

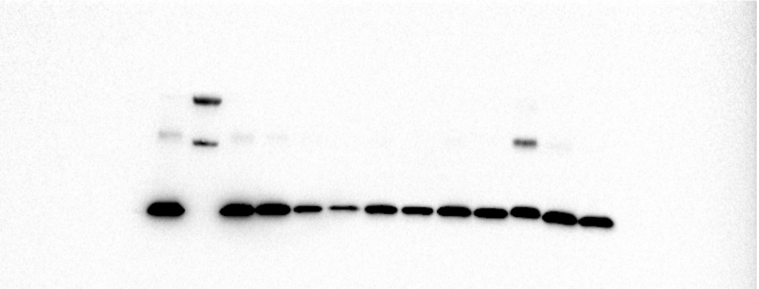

#30 T M T S T S T S T S ESR T S
34-2-1 34-20-1 34-5 34-6 34-7 34-8

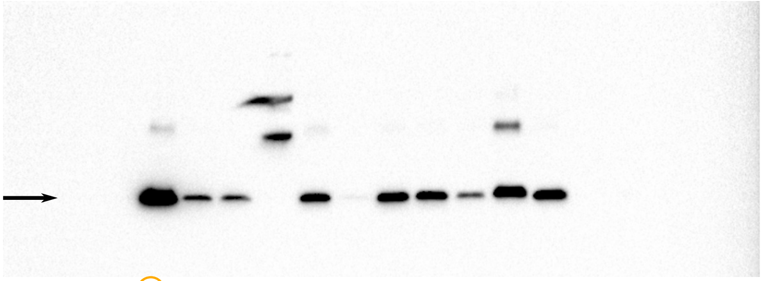

#11 M T T S T S T S T ESR T S T
24-1 24-2 24-3 24-5 24-10 24-6 24-11

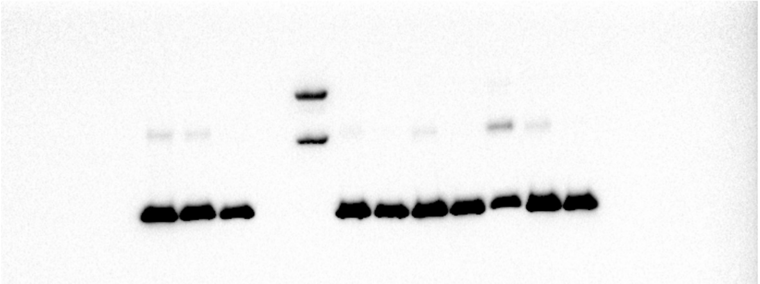

#12 T T S S M T S T S ESR T S S
24-1-2 24-4 24-10-1 24-7 24-8 24-9 24-11-1

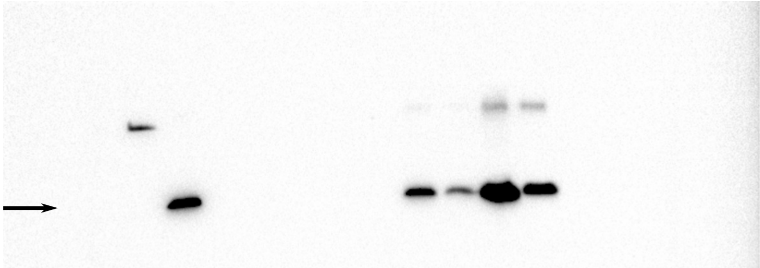

#13 M T S T S T S T S T ESR T S
25-1-1 25-2 25-3 25-4 27-1-2 25-5

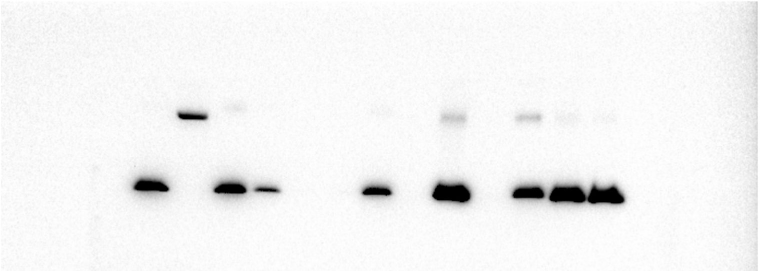

#14 T M T S T S T S T S ESR T S
25-1 25-6 25-7 25-8 27-1-1 27-12

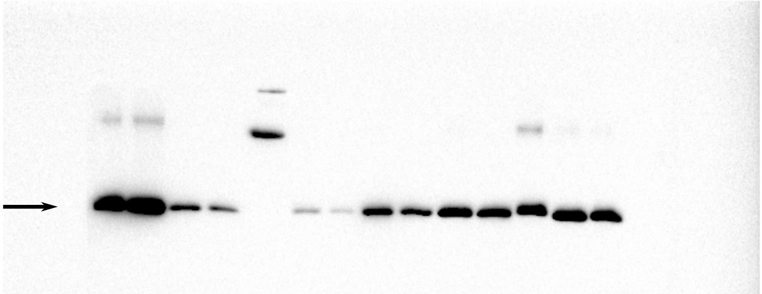

#15 T T S M T S T S T S ESR T S
27-1-3 27-2 27-3 27-4 27-5 27-10

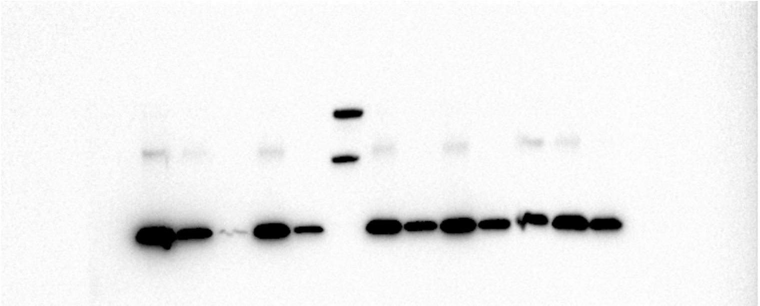

#16 T T S T S M T S T S ESR T S
27-1 27-6 27-7 27-8 27-9 27-11

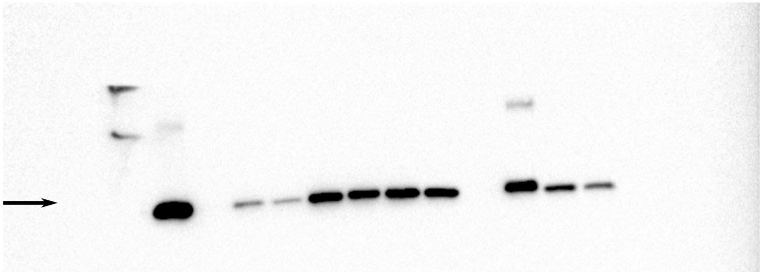

#17 M T S T S T S T S T ESR T S
26-1 26-2 26-3 26-4 26-11 26-5

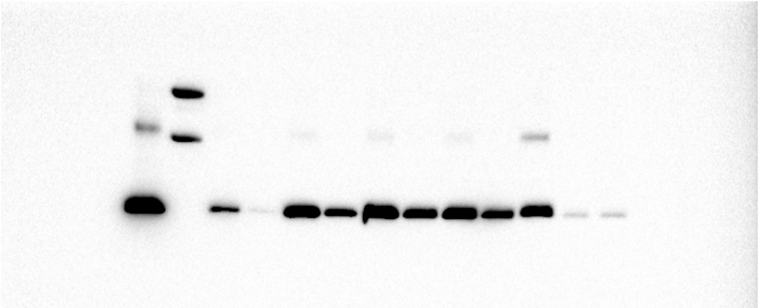

#18 T M T S T S T S T S ESR T S
26-1-1 26-6 26-7 26-8 26-9 26-10

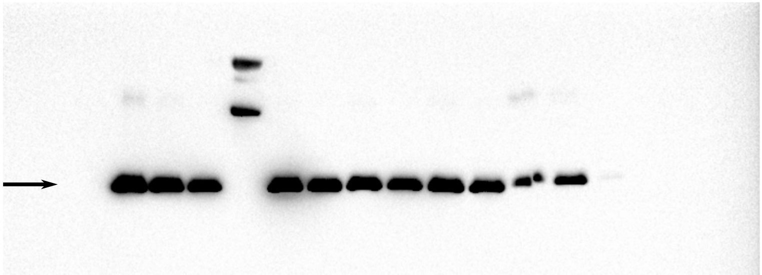

#19 T T S M T S T S T S ESR T S
28-1 28-10 28-11 28-12 28-2 28-6

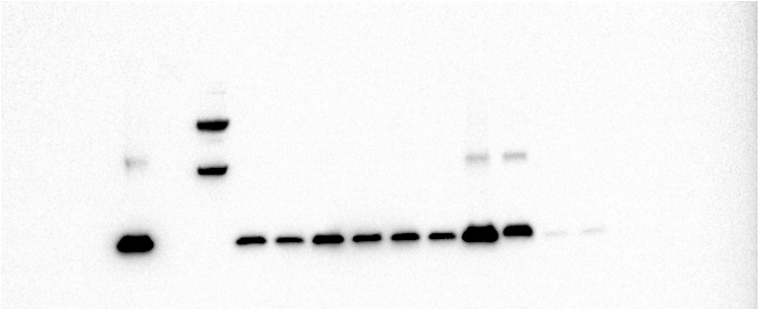

#20 T S M T S T S T S T ESR T S S
28-1-1 28-3 28-4 28-5 26-1-2 26-12 26-11-1

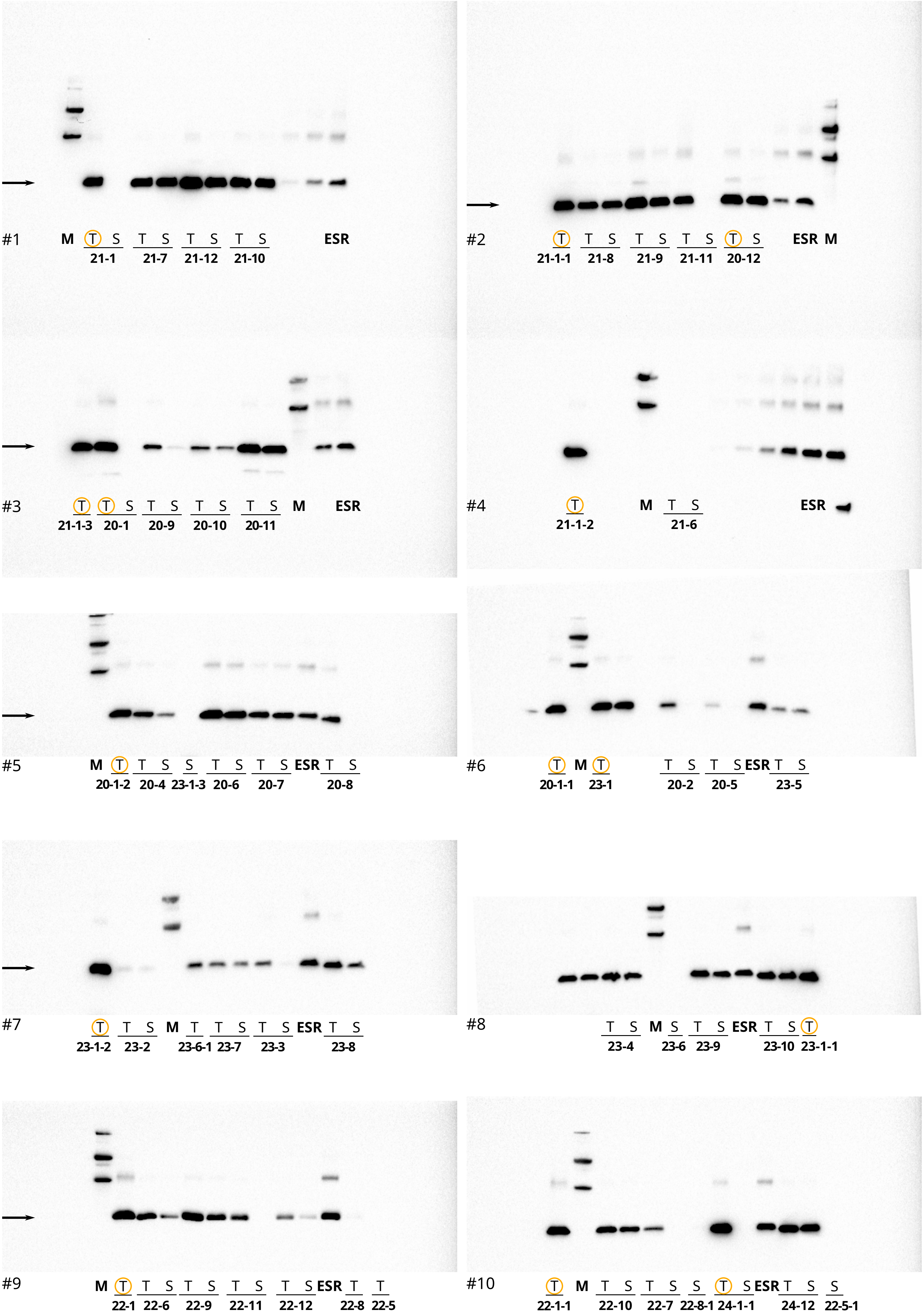

#1
M T S T S T S T S ESR
21-1 21-7 21-12 21-10
#2
T T S T S T S T S ESR M
21-1-1 21-8 21-9 21-11 20-12
#3
T T S T S T S T S M ESR
21-1-3 20-1 20-9 20-10 20-11
#4
T M T S ESR
21-1-2 21-6
#5
M T T S S T S T S ESR T S
20-1-2 20-4 23-1-3 20-6 20-7 20-8
#6
T M T T S T S ESR T S
20-1-1 23-1 20-2 20-5 23-5
#7
T T S M T T S T S ESR T S
23-1-2 23-2 23-6-1 23-7 23-3 23-8
#8
T S M S T S ESR T S T
23-4 23-6 23-9 23-10 23-1-1
#9
M T T S T S T S T S ESR T T
22-1 22-6 22-9 22-11 22-12 22-8 22-5
#10
T M T S T S S T S ESR T S S
22-1-1 22-10 22-7 22-8-1 24-1-1 24-12 22-5-1
