# Supplementary material for: Facade-Based Bicelles as a New Tool for Production of Active Membrane Proteins in a Cell-Free System
Source: Int J Mol Sci. 2023 Oct 3;24(19):14864. doi: 10.3390/ijms241914864 (PMC10573531; doi:10.3390/ijms241914864)
Supplement: Supplementary file 1 [file ijms-24-14864-s001.zip › GoncharukMV_Facades_CF-Supplementary.pdf]

Supplementary materials

Facade-based bicelles as a new tool for production of active membrane proteins in a cell-free system

Marina V. Goncharuk[1], Ekaterina V. Vasileva[1], Egor A. Ananiev[1], Andrey Y. Gorokhovatsky[1], Eduard V. Bocharov[1,2], Konstantin S. Mineev[1,†,‡] and Sergey A. Goncharuk[1,2,‡,*]

Goncharuk MV[1], Vasileva EV[1], Ananiev EA[1], Gorokhovatsky AY[1], Bocharov EV[1,2], Mineev KS[1,#,†], Goncharuk SA[1,2,†,*]

[1] Shemyakin-Ovchinnikov Institute of Bioorganic Chemistry of the Russian Academy of Sciences, Moscow, Russia.
[2] Moscow Institute of Physics and Technology, Dolgoprudny, Russia.
* Correspondence: ms.goncharuk@gmail.com.
[†] Current address: Goethe University Frankfurt, Frankfurt am Main, 60438, Germany.
[‡] These authors contributed equally to this work.

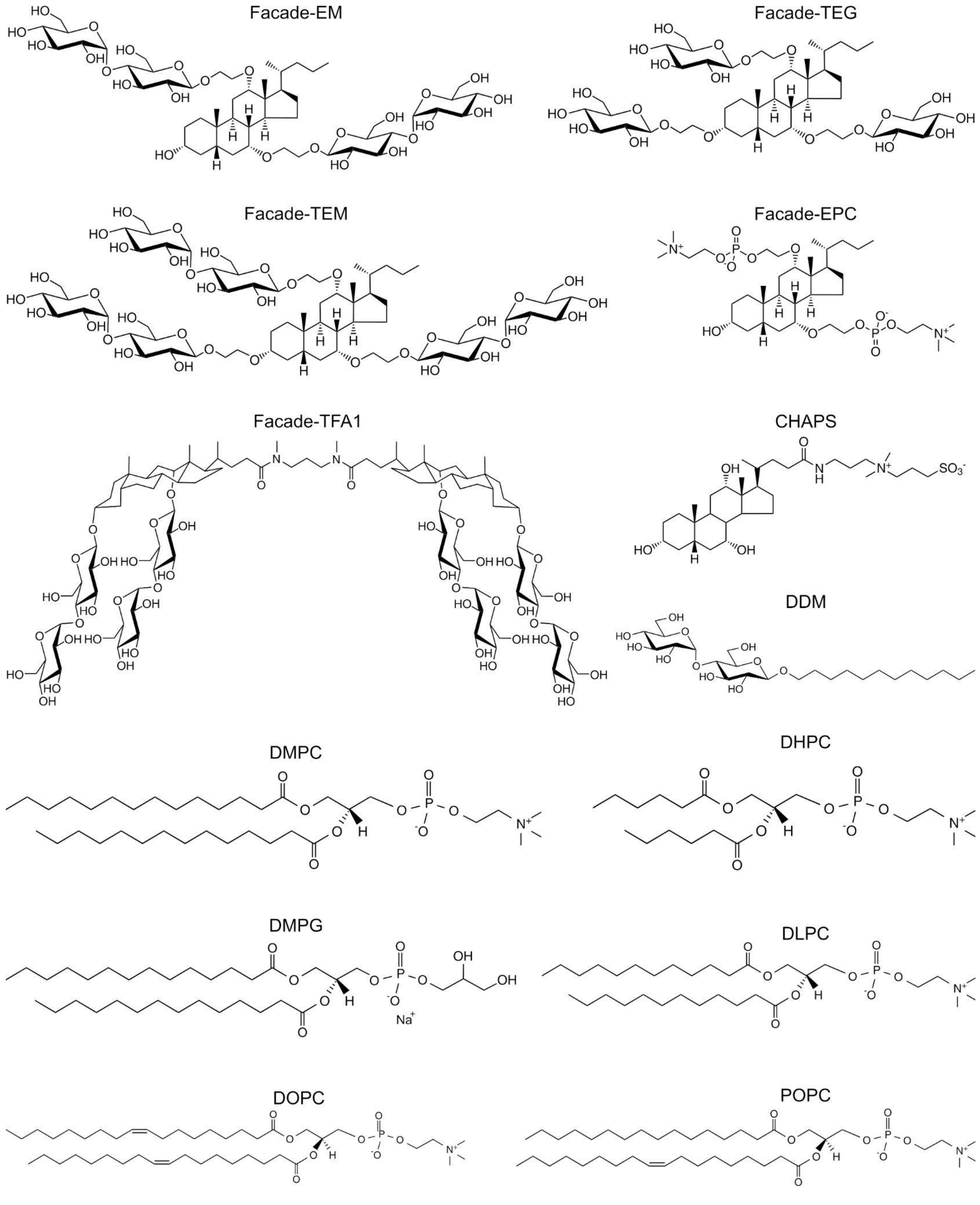

Supplementary Figure S1. The structures of the detergents and lipids used for cell-free protein synthesis.

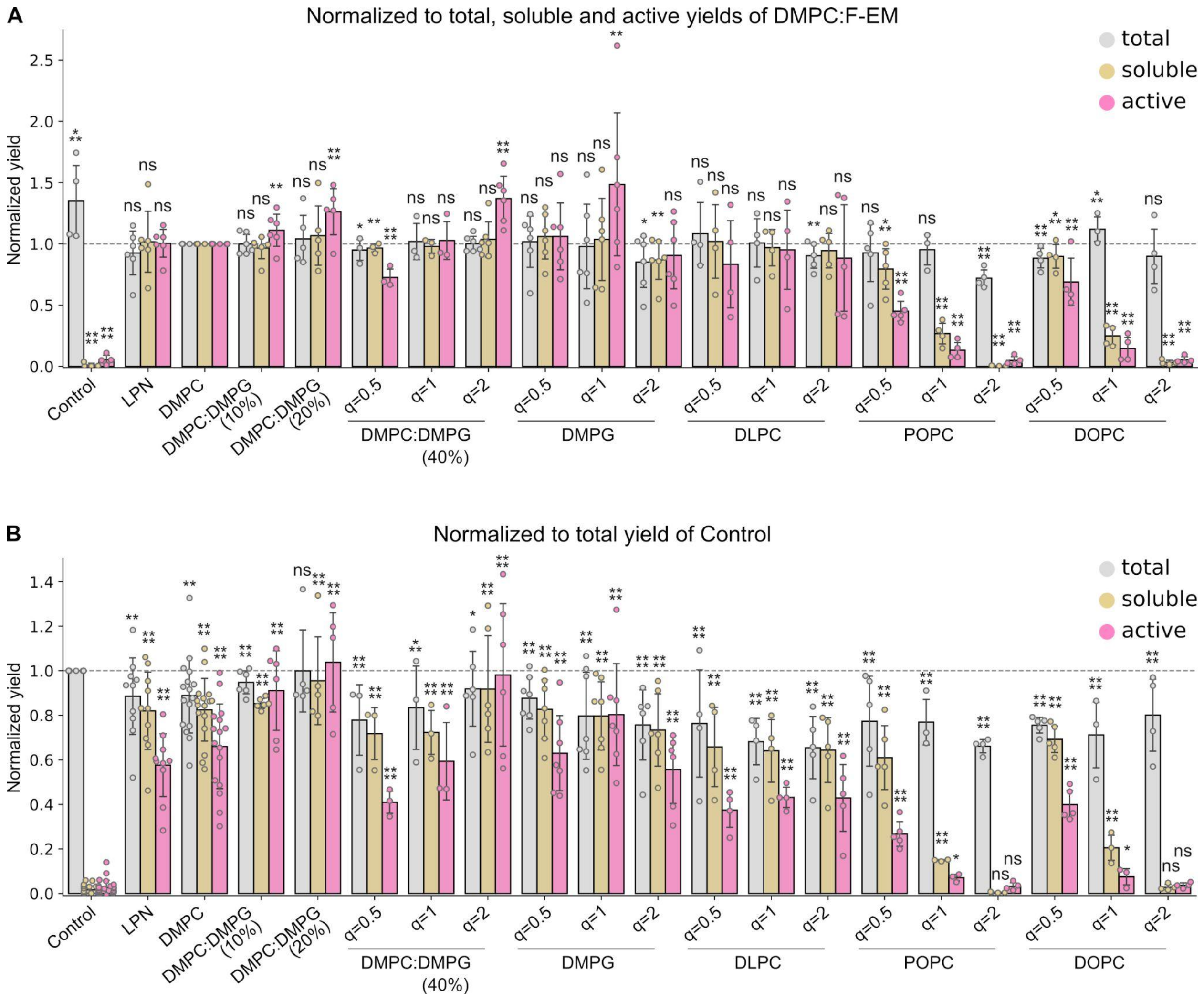

Supplementary Figure S2. The influence of lipids composition to cell-free expression levels of total, soluble and active ESR for Facade-EM bicelles. (**A**) The total, soluble, and active protein yields for all samples were normalized to the total, soluble, and active protein yields obtained for DMPC:F-EM bicelles at q=2, respectively. (**B**) All yields were normalized to the total yield of the control sample (cell-free reaction without any membrane mimetics). The total (gray) and soluble (yellow) expression levels were estimated by Western blot analysis. The yield of active (purple) ESR was quantified by UV-Vis spectroscopy. 15 mM of lipid in the RM and 0.1 mM of F-EM in FM was used for all bicelles samples. If otherwise not specified the q=2 was used. Error bars denote the standard deviations; statistical significance is provided according to a multiple t-test with Holm-Bonferroni correction (* - $p<0.05$, ** - $p<0.01$, *** - $p<0.001$, **** - $p<0.0001$, and ns denotes that changes are not significant). All statistical data are presented in Supplementary Table 5,6.

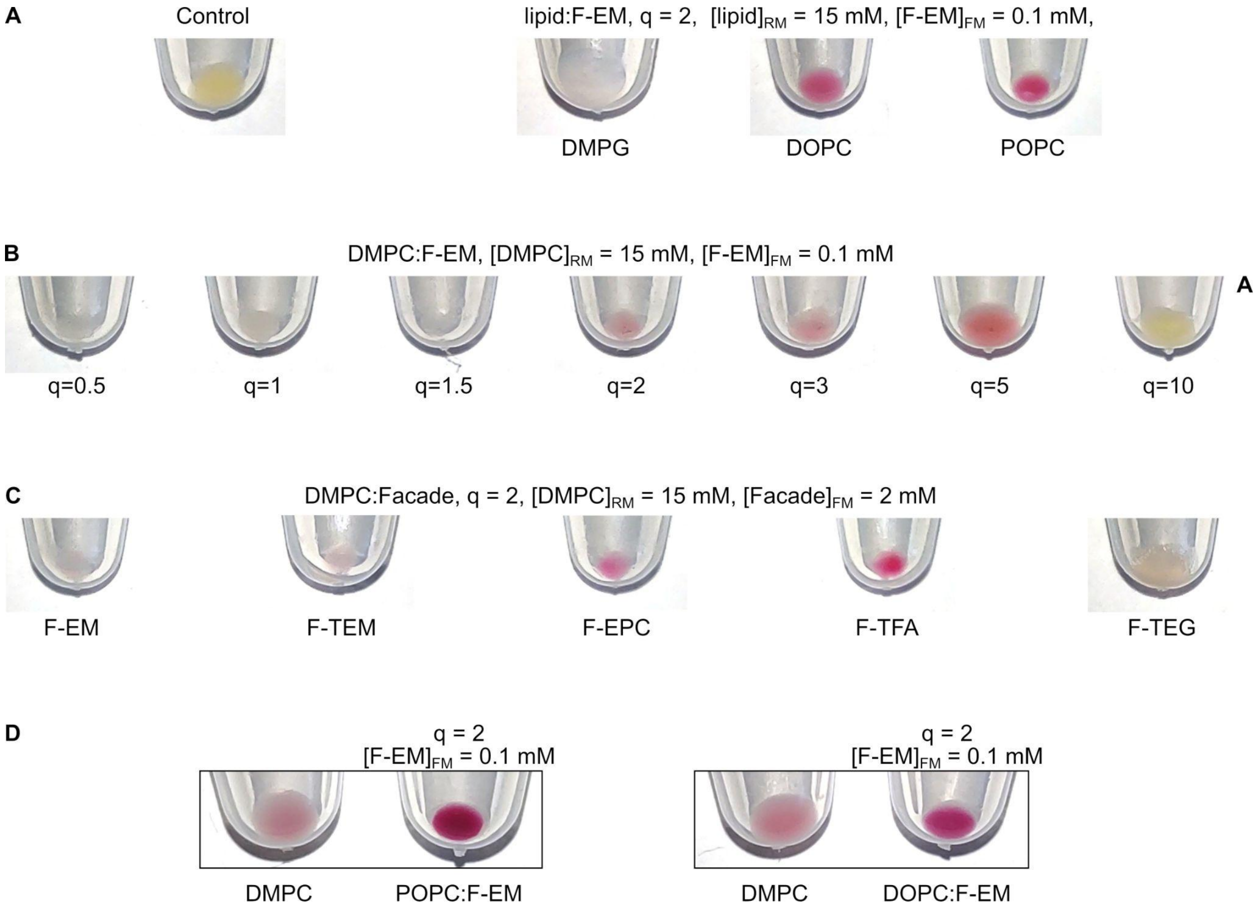

Supplementary Figure S3. Comparison of the precipitates after cell-free expression in different conditions. (**A**) - Comparison of different lipids in Facade-EM bicelles. (**B**) - The influence of q upon DMPC:F-EM bicelles. (**C**) - Comparison of different Facades in DMPC bicelles. (**D**) - The precipitates after cell-free synthesis in the presence of DMPC liposomes and POPC or DOPC bicelles in the same experiment and in the same photo.

Supplementary table S1. The statistical data for Figure 2. Statistical significance is provided according to a multiple t-test with Holm-Bonferroni correction. The values of total, soluble and active yields of the ESR in the Control sample were used to compare.

Membrane mimetic	fraction	p-value	t-statistic
DDM	total	5.770e-14	1.449e+01
	soluble	3.325e-02	2.248e+00
	active	9.172e-01	1.050e-01
CHAPS	total	1.805e-19	2.342e+01
	soluble	4.373e-01	7.885e-01
	active	2.509e-01	1.173e+00
DHPC	total	5.485e-63	9.803e+02
	soluble	8.076e-02	1.814e+00
	active	7.179e-01	3.651e-01
F-EPC	total	4.725e-38	1.444e+02
	soluble	6.243e-01	4.959e-01
	active	9.657e-02	1.727e+00
F-TFA	total	8.327e-01	2.135e-01
	soluble	1.899e-11	1.146e+01
	active	3.169e-01	1.021e+00
F-TEG	total	1.983e-19	2.554e+01
	soluble	8.342e-01	2.115e-01
	active	6.296e-02	1.946e+00
F-EM	total	2.652e-10	1.009e+01
	soluble	1.532e-07	7.196e+00
	active	9.906e-01	1.188e-02
F-TEM	total	5.488e-32	8.252e+01
	soluble	4.347e-23	3.609e+01
	active	4.512e-01	7.654e-01

DMPC:CHAPS	total	6.701e-16	1.555e+01
	soluble	1.830e-01	1.363e+00
	active	8.222e-01	2.267e-01
DMPC:DHPC	total	3.145e-22	2.523e+01
	soluble	6.901e-06	5.396e+00
	active	9.131e-05	4.493e+00
DMPC:F-EPC	total	2.180e-12	1.236e+01
	soluble	6.094e-11	1.061e+01
	active	1.498e-04	4.434e+00
DMPC:F-TFA	total	6.808e-06	5.661e+00
	soluble	3.432e-16	1.866e+01
	active	1.328e-08	8.252e+00
DMPC:F-TEG	total	1.023e-12	1.247e+01
	soluble	2.897e-01	1.080e+00
	active	5.320e-01	6.331e-01
DMPC:F-EM	total	6.171e-05	4.476e+00
	soluble	7.236e-22	1.929e+01
	active	6.376e-17	1.392e+01
DMPC:F-TEM	total	5.692e-03	3.025e+00
	soluble	7.597e-21	2.922e+01
	active	1.635e-15	1.745e+01
LPN	total	3.454e-03	3.158e+00
	soluble	9.523e-21	2.177e+01
	active	6.503e-18	1.746e+01
DMPC	total	3.030e-23	3.267e+01
	soluble	1.024e-01	1.691e+00
	active	3.104e-01	1.034e+00

Supplementary table S2. The statistical data for Figure 3A. Statistical significance is provided according to a multiple t-test with Holm-Bonferroni correction. The values of total, soluble and active yields of ESR in the presence of bicelles (DMPC:F-EM, DMPC:CHAPS or DMPC:DHPC) at q=0.5 were used to compare.

Membrane mimetic	q vaule	fraction	p-value	t-value
DMPC:F-EM	1	total	1.479e-01	1.495e+00
		soluble	5.502e-03	3.051e+00
		active	1.043e-07	7.468e+00
	1.5	total	3.211e-01	1.014e+00
		soluble	8.192e-02	1.819e+00
		active	9.387e-06	5.652e+00
	2	total	1.481e-01	1.484e+00
		soluble	1.422e-02	2.603e+00
		active	3.114e-09	8.272e+00
	3	total	4.729e-04	4.211e+00
		soluble	9.896e-01	1.322e-02
		active	1.522e-03	3.699e+00
	5	total	1.506e-12	1.614e+01
		soluble	4.201e-59	4.707e+03
		active	5.622e-50	1.557e+03
	10	total	8.967e-37	3.144e+02
		soluble	2.677e-46	9.972e+02
		active	8.843e-32	1.716e+02
DMPC:CHAPS	1	total	9.494e-04	4.621e+00
		soluble	3.373e-03	3.820e+00
		active	8.773e-05	6.312e+00
	1.5	total	1.673e-01	1.502e+00
		soluble	1.540e-02	2.982e+00

		active	3.750e-03	3.877e+00
	2	total	3.293e-02	2.517e+00
		soluble	6.197e-04	5.130e+00
		active	3.558e-05	7.535e+00
DMPC:DHPC	1	total	2.212e-02	2.597e+00
		soluble	1.586e-02	2.772e+00
		active	3.878e-02	2.298e+00
	1.5	total	3.027e-02	2.486e+00
		soluble	5.224e-03	3.472e+00
		active	9.958e-04	4.440e+00
	2	total	3.955e-01	8.842e-01
		soluble	2.589e-01	1.191e+00
		active	2.037e-02	2.708e+00

Supplementary table S3. The statistical data for Figure 3B. Statistical significance is provided according to a multiple t-test with Holm-Bonferroni correction. The values of total, soluble and active yields of the ESR in the Control sample were used to compare.

Membrane mimetic	q vaule	fraction	p-value	t-value
LPN	-	total	3.454e-03	3.158e+00
		soluble	9.523e-21	2.177e+01
		active	6.503e-18	1.746e+01
DMPC:F-EM	0.5	total	6.171e-05	4.476e+00
		soluble	7.236e-22	1.929e+01
		active	6.376e-17	1.392e+01
	1	total	9.097e-01	1.143e-01
		soluble	4.484e-25	3.142e+01
		active	6.636e-17	1.650e+01
	1.5	total	2.259e-04	4.209e+00
		soluble	9.907e-20	2.217e+01
		active	2.042e-12	1.160e+01
	2	total	3.263e-03	3.140e+00
		soluble	1.791e-26	2.712e+01
		active	4.234e-18	1.559e+01
	3	total	9.731e-09	8.391e+00
		soluble	1.991e-17	2.106e+01
		active	2.985e-13	1.388e+01
	5	total	4.027e-19	2.480e+01
		soluble	1.950e-01	1.332e+00
		active	5.381e-01	6.243e-01
	10	total	8.485e-51	4.675e+02
		soluble	2.788e-01	1.107e+00

		active	9.890e-01	1.395e-02
DMPC:CHAPS	0.5	total	6.701e-16	1.555e+01
		soluble	1.830e-01	1.363e+00
		active	8.222e-01	2.267e-01
	1	total	5.804e-03	2.996e+00
		soluble	1.195e-11	1.119e+01
		active	1.222e-06	6.209e+00
	1.5	total	1.534e-12	1.255e+01
		soluble	7.666e-10	9.394e+00
		active	6.690e-07	6.510e+00
	2	total	1.208e-13	1.404e+01
		soluble	8.744e-21	2.759e+01
		active	1.614e-08	8.039e+00
DMPC:DHPC	0.5	total	3.145e-22	2.523e+01
		soluble	6.901e-06	5.396e+00
		active	9.131e-05	4.493e+00
	1	total	4.664e-16	1.618e+01
		soluble	9.838e-09	7.919e+00
		active	3.797e-07	6.527e+00
	1.5	total	2.027e-20	2.548e+01
		soluble	3.861e-18	2.078e+01
		active	1.626e-09	8.900e+00
	2	total	4.662e-15	1.564e+01
		soluble	2.007e-07	6.909e+00
		active	2.015e-06	6.018e+00

Supplementary table S4. The statistical data for Figure 4. Statistical significance is provided according to a multiple t-test with Holm-Bonferroni correction. The values of total, soluble and active yields of ESR in the presence of DMPC:F-EM bicelles at the 0.1 mM of F-EM concentration in FM were used to compare.

q vaule	F-EM concentration In FM	fraction	p-value	t-value
0.5 (30 mM F-EM in RM)	1	total	1.323e-03	3.761e+00
		soluble	3.052e-03	3.393e+00
		active	2.298e-04	4.529e+00
	10	total	8.074e-02	1.845e+00
		soluble	1.257e-02	2.756e+00
		active	4.239e-01	8.173e-01
	30	total	2.048e-01	1.313e+00
		soluble	1.313e-01	1.577e+00
		active	2.422e-04	4.505e+00
2 (7.5 mM F-EM in RM)	1	total	2.139e-02	2.520e+00
		soluble	6.372e-01	4.797e-01
		active	7.067e-02	1.921e+00
	2	total	9.239e-01	9.678e-02
		soluble	8.808e-01	1.519e-01
		active	8.053e-02	1.841e+00
	7.5	total	9.566e-01	5.524e-02
		soluble	9.068e-01	1.189e-01
		active	1.708e-06	7.123e+00

Supplementary table S5. The statistical data for Figure 5 and Supplementary Figure S2A. Statistical significance is provided according to a multiple t-test with Holm-Bonferroni correction. The values of total, soluble and active yields of ESR in the presence of DMPC:F-EM bicelles were used to compare.

Lipid composition of the F-EM bicelles	q value	fraction	p-value	t-value
Control sample	-	total	2.345e-04	4.576e+00
		soluble	1.986e-33	2.533e+02
		active	2.804e-26	1.014e+02
LPN	-	total	1.279e-01	1.588e+00
		soluble	7.961e-01	2.619e-01
		active	8.760e-01	1.580e-01
DMPC:DMPG (10:1)	2	total	9.729e-01	3.436e-02
		soluble	1.598e-01	1.460e+00
		active	4.159e-03	3.234e+00
DMPC:DMPG (5:1)	2	total	4.222e-01	8.204e-01
		soluble	3.016e-01	1.062e+00
		active	3.937e-05	5.317e+00
DMPC:DMPG (2.5:1)	0.5	total	3.999e-02	2.224e+00
		soluble	1.391e-03	3.813e+00
		active	2.426e-11	1.523e+01
	1	total	6.258e-01	4.967e-01
		soluble	1.922e-01	1.358e+00
		active	5.063e-01	6.790e-01
	2	total	9.476e-01	6.652e-02
		soluble	3.427e-01	9.720e-01
		active	1.818e-07	7.772e+00
DMPG	0.5	total	7.487e-01	8.222e-01
		soluble	2.261e-01	1.249e+00

		active	4.040e-01	8.526e-01
	1	total	8.222e-01	2.277e-01
		soluble	6.840e-01	4.130e-01
		active	4.778e-03	3.173e+00
	2	total	1.266e-02	2.739e+00
		soluble	5.216e-03	3.135e+00
		active	2.034e-01	1.315e+00
DLPC	0.5	total	2.299e-01	1.243e+00
		soluble	8.045e-01	2.512e-01
		active	9.329e-02	1.772e+00
	1	total	8.858e-01	1.457e-01
		soluble	4.469e-01	7.775e-01
		active	5.782e-01	5.663e-01
	2	total	1.565e-03	3.687e+00
		soluble	1.459e-01	1.516e+00
		active	3.248e-01	1.011e+00
POPC	0.5	total	2.496e-01	1.188e+00
		soluble	1.522e-04	4.711e+00
		active	3.427e-16	2.560e+01
	1	total	1.675e-01	1.442e+00
		soluble	1.406e-17	3.310e+01
		active	4.119e-21	5.226e+01
	2	total	4.931e-12	1.588e+01
		soluble	1.862e-51	2.543e+03
		active	2.296e-27	1.166e+02
DOPC	0.5	total	2.802e-05	5.561e+00
		soluble	8.118e-04	4.015e+00

		active	1.013e-05	6.052e+00
	1	total	2.888e-04	4.542e+00
		soluble	1.042e-18	3.833e+01
		active	4.304e-18	3.539e+01
	2	total	9.983e-02	1.735e+00
		soluble	1.586e-32	2.257e+02
		active	7.088e-29	1.415e+02

Supplementary table S6. The statistical data for Supplementary Figure S2B. Statistical significance is provided according to a multiple t-test with Holm-Bonferroni correction. The values of total, soluble and active yields of the ESR in the Control sample were used to compare.

Lipid composition of the F-EM bicelles	q value	fraction	p-value	t-value
LPN	-	total	3.454e-03	3.158e+00
		soluble	9.523e-21	2.177e+01
		active	6.503e-18	1.746e+01
DMPC	2	total	3.263e-03	3.140e+00
		soluble	1.791e-26	2.712e+01
		active	4.234e-18	1.559e+01
DMPC:DMPG (10:1)	2	total	4.601e-05	4.815e+00
		soluble	8.442e-37	9.590e+01
		active	3.087e-19	2.205e+01
DMPC:DMPG (5:1)	2	total	9.838e-01	2.051e-02
		soluble	8.296e-19	2.207e+01
		active	5.929e-18	2.043e+01
DMPC:DMPG (2.5:1)	0.5	total	6.536e-07	6.597e+00
		soluble	1.164e-19	2.611e+01
		active	1.777e-15	1.739e+01
	1	total	3.095e-04	4.183e+00
		soluble	3.524e-21	3.016e+01
		active	4.742e-13	1.359e+01
	2	total	3.007e-02	2.285e+00
		soluble	1.057e-16	1.764e+01
		active	4.668e-14	1.386e+01
DMPG	0.5	total	8.555e-07	6.227e+00
		soluble	1.523e-22	2.800e+01

		active	6.489e-16	1.598e+01
	1	total	3.143e-05	4.923e+00
		soluble	1.639e-20	2.367e+01
		active	1.275e-15	1.557e+01
	2	total	5.519e-08	7.333e+00
		soluble	2.306e-18	2.043e+01
		active	4.936e-15	1.517e+01
DLPC	0.5	total	8.705e-05	4.640e+00
		soluble	2.576e-15	1.654e+01
		active	3.412e-14	1.482e+01
	1	total	6.661e-14	1.440e+01
		soluble	2.355e-17	2.008e+01
		active	7.735e-18	2.101e+01
	2	total	4.465e-12	1.169e+01
		soluble	1.429e-17	1.973e+01
		active	7.123e-12	1.146e+01
POPC	0.5	total	1.368e-05	5.298e+00
		soluble	4.235e-17	1.890e+01
		active	6.971e-13	1.268e+01
	1	total	8.674e-11	1.066e+01
		soluble	2.511e-12	1.260e+01
		active	3.380e-02	2.246e+00
	2	total	3.337e-28	5.386e+01
		soluble	2.146e-01	1.272e+00
		active	7.382e-01	3.379e-01
DOPC	0.5	total	2.792e-23	3.277e+01
		soluble	5.831e-27	4.511e+01

		active	6.349e-17	1.860e+01
	1	total	1.751e-09	9.180e+00
		soluble	9.054e-12	1.187e+01
		active	2.961e-02	2.307e+00
	2	total	3.861e-06	5.826e+00
		soluble	2.252e-01	1.242e+00
		active	5.493e-01	6.067e-01

Supplementary table S7. The statistical data for Figure 6A. Statistical significance is provided according to a multiple t-test with Holm-Bonferroni correction. The values of total, soluble and active yields of ESR in the presence of DMPC:F-EM bicelles were used to compare.

Membrane mimetic	fraction	p-value	t-value
DMPC:F-TEM	total	3.750e-03	4.585e+00
	soluble	2.689e-03	4.908e+00
	active	1.594e-04	8.358e+00
DMPC:F-TFA	total	1.616e-01	1.596e+00
	soluble	9.228e-05	9.213e+00
	active	1.317e-08	4.146e+01
DMPC:F-EPC	total	1.073e-01	1.892e+00
	soluble	6.519e-03	4.078e+00
	active	1.410e-02	3.423e+00
DMPC:F-TEG	total	5.261e-08	3.288e+01
	soluble	4.471e-20	3.387e+03
	active	1.520e-10	8.732e+01
LPN	total	9.829e-01	2.240e-02
	soluble	8.615e-01	1.821e-01
	active	7.721e-01	3.031e-01
Control	total	1.141e-01	1.848e+00
	soluble	3.831e-12	1.613e+02
	active	5.352e-11	1.039e+02

Supplementary table S8. The statistical data for Figure 6B. Statistical significance is provided according to a multiple t-test with Holm-Bonferroni correction. The values of total, soluble and active yields of the ESR in the presence of DMPC:F-EM bicelles were used to compare.

Membrane mimetic	fraction	p-value	t-value
DMPC:F-TEM	total	1.493e-01	1.654e+00
	soluble	1.040e-01	1.915e+00
	active	4.837e-03	4.347e+00
DMPC:F-TFA	total	6.796e-01	4.339e-01
	soluble	8.486e-03	3.847e+00
	active	9.808e-05	9.114e+00
DMPC:F-EPC	total	4.987e-01	7.199e-01
	soluble	3.700e-01	9.689e-01
	active	1.503e-02	3.370e+00
DMPC:F-TEG	total	1.197e-03	5.757e+00
	soluble	5.322e-04	6.710e+00
	active	3.825e-05	1.075e+01
LPN	total	8.651e-01	1.773e-01
	soluble	9.181e-01	1.073e-01
	active	8.690e-01	1.722e-01
Control	total	9.319e-01	8.914e-02
	soluble	5.858e-04	6.591e+00
	active	3.595e-05	1.087e+01
